# A meta-analysis on the risk factors adjusted association between cardiovascular disease and COVID-19 severity

**DOI:** 10.1186/s12889-021-11051-w

**Published:** 2021-08-11

**Authors:** Jie Xu, Wenwei Xiao, Xuan Liang, Li Shi, Peihua Zhang, Ying Wang, Yadong Wang, Haiyan Yang

**Affiliations:** 1 Department of Epidemiology, School of Public Health, Zhengzhou University, No. 100 of Science Avenue, Zhengzhou, 450001 China; 2 Department of Toxicology, Henan Center for Disease Control and Prevention, Zhengzhou, 450016 China

**Keywords:** Coronavirus disease 2019, cardiovascular disease, adverse outcome, adjusted effect estimate

## Abstract

**Background:**

Cardiovascular disease (CVD), one of the most common comorbidities of coronavirus disease 2019 (COVID-19), has been suspected to be associated with adverse outcomes in COVID-19 patients, but their correlation remains controversial.

**Method:**

This is a quantitative meta-analysis on the basis of adjusted effect estimates. PubMed, Web of Science, MedRxiv, Scopus, Elsevier ScienceDirect, Cochrane Library and EMBASE were searched comprehensively to obtain a complete data source up to January 7, 2021. Pooled effects (hazard ratio (HR), odds ratio (OR)) and the 95% confidence intervals (CIs) were estimated to evaluate the risk of the adverse outcomes in COVID-19 patients with CVD. Heterogeneity was assessed by Cochran’s Q-statistic, I^2^test, and meta-regression. In addition, we also provided the prediction interval, which was helpful for assessing whether the variation across studies was clinically significant. The robustness of the results was evaluated by sensitivity analysis. Publication bias was assessed by Begg’s test, Egger’s test, and trim-and-fill method.

**Result:**

Our results revealed that COVID-19 patients with pre-existing CVD tended more to adverse outcomes on the basis of 203 eligible studies with 24,032,712 cases (pooled ORs = 1.41, 95% CIs: 1.32-1.51, prediction interval: 0.84-2.39; pooled HRs = 1.34, 95% CIs: 1.23-1.46, prediction interval: 0.82-2.21). Further subgroup analyses stratified by age, the proportion of males, study design, disease types, sample size, region and disease outcomes also showed that pre-existing CVD was significantly associated with adverse outcomes among COVID-19 patients.

**Conclusion:**

Our findings demonstrated that pre-existing CVD was an independent risk factor associated with adverse outcomes among COVID-19 patients.

**Supplementary Information:**

The online version contains supplementary material available at 10.1186/s12889-021-11051-w.

## Introduction

Since December 2019, the severe acute respiratory syndrome coronavirus 2 (SARS-CoV-2) has caused a global outbreak of coronavirus disease 2019 (COVID-19). Currently, the pandemic has affected more than 127,319,002 people in more than 200 countries and killed more than 2,785,838 people (https://www.who.int/emergencies/diseases/novel-coronavirus-2019). Previous studies have reported that several pre-existing medical conditions, such as hypertension, diabetes and so on, might accelerate disease progression of COVID-19 [1–3]. Cardiovascular disease (CVD), one of the most common comorbidities of COVID-19, has been observed to be associated with adverse outcomes among COVID-19 patients by Li et al. in a meta-analysis study [4]. Nevertheless, it is worth noting that the results of Li et al.’s study were based on the unadjusted effect estimates [4]. It is reported that age, sex, and co-existing diseases are known to affect the outcomes of COVID-19 patients [5–7], which may modulate the association between CVD and adverse outcomes in COVID-19 patients. Moreover, Zhou et al. observed that coronary heart disease (CHD), one of CVD, was strongly correlated with an increased risk of in-hospital mortality among COVID-19 patients in univariable analysis (odds ratio (OR) = 21.4, 95% confidence interval (CI): 4.64-98.76), but no significant correlation was observed in multivariable analysis (OR = 2.14, 95% CI: 0.26-17.79) [8]. The similar results were also observed by Robilotti et al. [9] and Louapre et al. [10]. Therefore, it is necessary to clarify whether pre-existing CVD was an independent risk factor associated with adverse outcomes in COVID-19 patients. In this study, we performed a quantitative meta-analysis on the basis of adjusted effect estimates.

## Methods

**Fig. 1:**
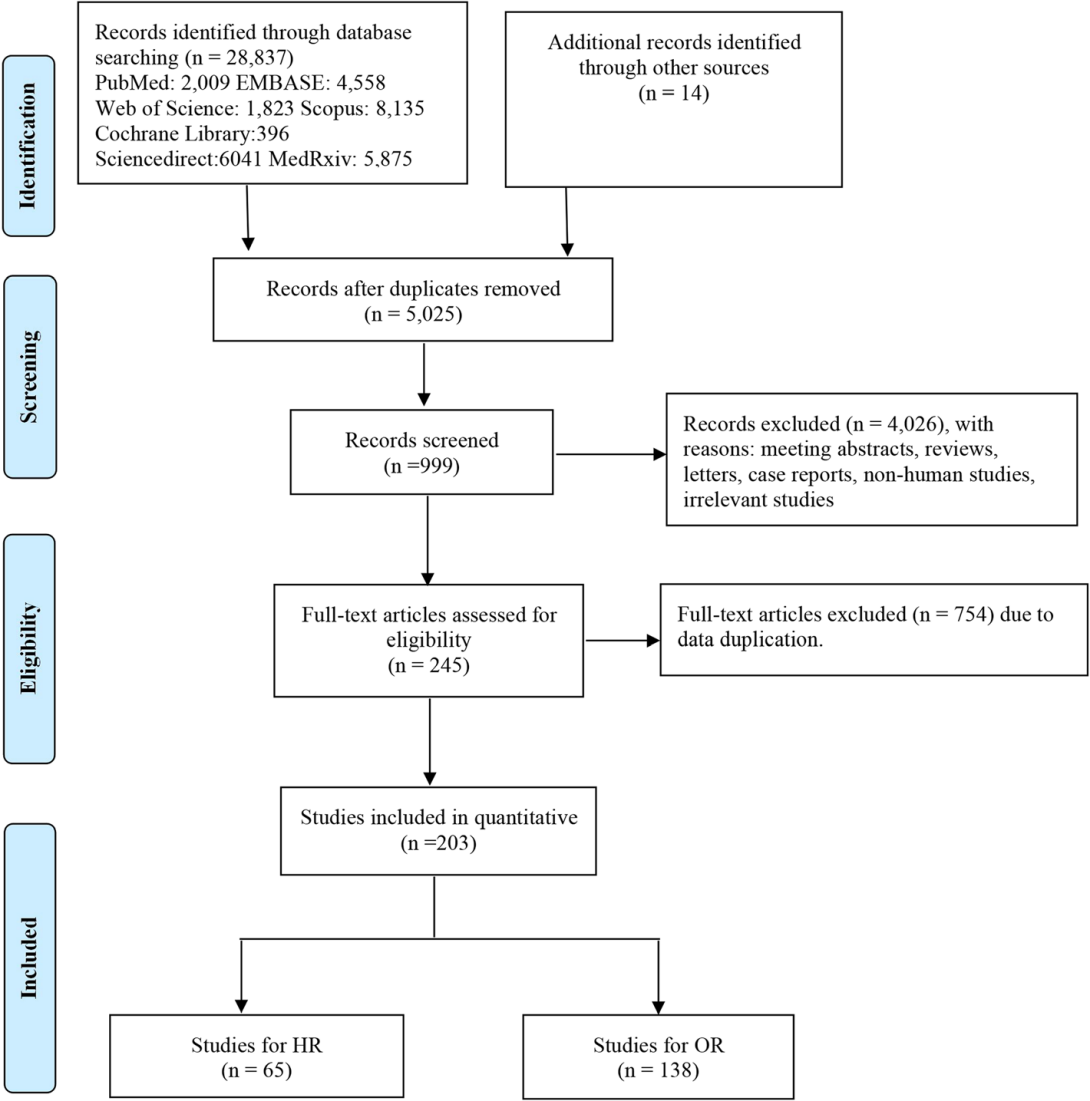
Flow diagram of selection process

This is a quantitative meta-analysis on the basis of adjusted effect estimates. Admittedly, our study was not registered, but our meta-analysis was made in strict accordance with the process of systematic evaluation (Fig. 1). Moreover, our study is less likely to be biased by artificial bias because this study was carried out rigorously in accordance with the Preferred Reporting Items for Systematic Reviews and Meta-analysis (PRISMA) guidelines (Online supplemental Table A1) [11].

### Literature search strategy

The databases of PubMed, Web of Science, MedRxiv, Scopus, Elsevier ScienceDirect, Cochrane Library and Embase were searched to obtain a complete data source up to January 7, 2021. The search strategies were as follows: (“COVID-19” OR “coronavirus disease 2019” OR “SARS-CoV-2” OR “2019-nCoV”) AND (“cardiovascular disease” OR “coronary heart disease” OR “cardiac disease” OR “heart disease” OR “heart failure” OR “coronary artery disease”) AND (“outcome” OR “severe” OR “critical” OR “severity” OR “fatality” OR “mortality” OR “death” OR “adverse outcome” OR “poor outcome” OR “clinical characteristics”). All the terms matched the MesH browser. Beyond that, the relevant references of preceding studies were also taken into account.

### Eligibility criteria

The criteria for including studies were: (1) Subjects should be laboratory-confirmed COVID-19 patients; (2) Studies should report the correlation between CVD and COVID-19 patients and the data are available; (3) Studies should be published in English; (4) Studies should include the multivariate analysis. The studies with the largest sample size were selected for inclusion when studies were conducted in the same hospital and the overlapping period. There was no restriction for region of study. The exclusion criteria included case reports, review papers, comments, errata, repeated studies, studies only reporting the characteristics of COVID-19 patients with CVD, and studies without available full text.

### Data extraction and quality assessment

Data were extracted independently by two investigators (J.X. and W.X.), including the following information: the first author, source of data, country, date of data collection, number of patients, mean/median age, the percent of males, study design, the percent of COVID-19 patients with CVD, adjusted effect estimates (hazard ratio (HR) or OR) and adjusted risk factors. When both OR and HR existed in the same article, it was preferred to include HR because cox regression took time into account. Two researchers negotiated to resolve it in case of any issues not covered by the criteria and Y.W. acted as arbiter. The quality of the included studies was evaluated by investigators according to the Newcastle-Ottawa Scale [12]. High-quality studies referred to studies with a score above 7.

### Data synthesis

The major information such as study design and effect estimates were directly extracted from original articles. The research type of some articles was not clear and some articles provided both OR and HR. Besides, the calculation methods of HR and OR are different. The calculation of HR takes into account the concept of time, and OR is the approximate value of risk ratio. Therefore, pooled HR, OR and 95% confidence intervals (CIs) were separately calculated to address the risk of adverse outcomes in COVID-19 patients with a history of CVD. Heterogeneity was assessed by Cochran’s Q-statistic and I^2^ test, if no significant heterogeneity was observed (I^2^ ≤ 50%, *P* > 0.1), a fixed-effects model was adopted; otherwise, a random-effects model was applied [13]. In addition, we also provided the prediction interval, which was helpful for assessing whether the variation across studies was clinically significant [14, 15]. The robustness of the results was evaluated by sensitivity analysis which omitted one study at a time. Publication bias was assessed by Begg’s test [16], Egger’s test [17] and trim-and-fill method [18]. Subgroup analysis and meta-regression were conducted to determine the source of heterogeneity. Data analyses were conducted using Stata, version 12.0 (meta-program) and R, version 3.6.1 (netmeta package). A two-tailed P-value < 0.05 was regarded as significant.

## Results

The flow chart of selection process is shown in Fig. 1. 5,025 records were retrieved after removing 23,826 duplicates, of which 245 studies were full-text assessed. Eventually, a total of 203 eligible studies with 24,032,712 patients were enrolled in our meta-analysis [2, 3, 8, 9, 19–210, 212–218]. 81 studies originated from Europe, 54 studies came from North America, 61 from Asia, 2 from Australia, and the remained 5 were not just from one country (Table 1). Among these studies, cardiac disease was mentioned in 63 studies, HF was involved in 35 studies, and CAD was involved in 35 studies (Table 2). Adjusted HR was reported in 65 studies and adjusted OR was reported in 138 studies (Table 2). The main characteristics of the selected studies are summarized in Table 1.

**Table 1:** Main characteristics of the included studies

Author (Year)	Country	Patients(n)	Mean/Median Age(years)	Male (%)	Study design	Kinds of diseases	CVD (%)	Adjusted effect estimate (95%CI)	Outcome	Confounders	NOS Score
Zhou et al. (2020) [8]	China	191	56·0 (46·0–67·0)	119 (62)	Retrospective cohort study	Coronary heart disease	18 (8)	OR 2.14 (0.26-17.79)	In-hospital death	Age, SOFA score	7
Yu et al. (2020) [19]	China	333	50(35-63)	172 (51.7)	Descriptive study	Heart disease	24 (7.2)	OR 4.2 (1.2-14.2)	Severity	Age, sex, diabetes, HTN, respiratory disease	8
Cummings et al. (2020) [3]	USA	257	62 (51–72)	171 (67)	Prospective observational cohort study t	Chronic cardiac disease	49 (19)	HR 1.76 (1.08-2.86)	In-hospital mortality	Age, gender, symptom duration before hospital, presentation, COPD or interstitial lung disease, diabetes, IL-6, D-dimer	8
Zhao et al. (2020) [20]	China	1000	61 (46-70)	466 (46.6)	Retrospective study	Coronary heart disease	60 (6)	HR 0.972 (0.547-1.726)	Death	Age	8
Sabri et al. (2020) [21]	Iran	60	54.1±15.5	NR	Retrospective cohort study	Heart Disease	10 (15.9)	OR 1.12 (1.08-1.14)	ICU admission	Pericardial effusion, blood oxygen saturation	7
Lala et al. (2020) [22]	USA	2736	66.4	1630 (59.6)	NR	Coronary Artery Disease	453 (16.6)	OR 1.08 (0.85-1.37)	Mortality	age, sex, BMI, race, ethnicity, history of CAD, history of AF, history of HF, history of HTN, history of CKD, history of DM, statin use, angiotensin converting enzyme inhibitor (ACEi) or angiotensin II receptor blocker (ARB) use, and CURB-65 score at hospital admission	7
Cen et al. (2020) [2]	China	1007	61(49-68)	493(49.0)	Multi-center observational study	Coronary artery disease	65 (6.5)	HR 1.828 (1.256-2.660)	Disease progression was defined as progression to the severe or critical disease stage, or death	Age, sex, smoking history, HTN, diabetes, chronic obstructive lung disease, CAD, CRD, CVA, hepatitis B infection, anti-viral drug, aeration of anti-viral therapy	7
Ciceri et al. (2020) [23]	Italy	410	65 (56-75)	299 (72.9)	NR	Coronary artery disease	51 (12.6)	HR 2.93 (1.77-4.86)	Death	Age, gender, cancer, radiographic assessment of lung edema score, WBC count, lymphocyte count, hemoglobin, platelets.	7
Barman et al. (2020) [24]	Turkey	607	59.5±14.8	334 (55.02)	Multi-center retrospective study	Coronary artery disease	116 (19.1)	OR 1.26 (1.06-1.50)	Mortality	Age, gender, HTN, diabetes, CAD, COPD, smoking, creatinine, uric acid, glucose	7
Bravi et al. (2020) [25]	Italy	1603	58.0±20.9	758 (47.36)	Case-control, retrospective study	Major cardiovascular diseases	258 (16.1)	OR 1.88 (1.32-2.70)	Severe or very severe/lethal	Age, gender, HTN, diabetes, cancer, COPD, renal disease	7
Deiana et al. (2020) [26]	Italy	1223	80.4±10.6	499 (40.8)	Matched case-control study	CVD	63 (64.9)	OR 4.0 (1.7-9.7)	Severity	Active tumors, diabetes, HIV, CLD, CRD, metabolic diseases, obesity, chronic neurological diseases, other pathologies	7
Zhang et al. (2020) [27]	China	80	51.16±17.476	33 (41.25)	Retrospective cohort	Cardiac disease	9 (11.25)	HR 0.21 (0-22.09)	Severity	Age, respiratory diseases, HTN, more than 2 kinds of diseases, WBC, neutrophil, LYM%, NEU%, NLR, FIB, CRP, TBIL, ALB, GFR, CK-MB, myoglobin, troponin	7
Nie et al. (2020) [28]	China	671	43±15.09	377 (56.2)	NR	CVD	70 (10.4)	OR 0.809 (0.306–2.142)	Severity	Age, gender, coexisting disorder (HTN, diabetes, respiratory diseases, diabetes, respiratory diseases), Animal/human transmission source contact, Contact with confirmed cases, Contact with confirmed cases, Contact with individuals who had been to Wuhan, Close to cluster outbreak, Visited hospital, Visited wet market, No contact, Days from illness onset to diagnosis, X-ray with pneumonia features, CT with pneumonia features, Blood routine test Leucocyte count, Lymphocyte count, Lymphocyte percentage, Neutrophil percentage	7
Robilotti et al. (2020) [9]	USA	423	60.2	212 (50)	NR	Cardiac disorder	84 (20)	HR 1.44 (0.88-2.37)	Severe respiratory illness,	Age, gender, race, BMI, smoking, asthma/COPD, cancer, major surgery, diabetes, HTN/CKI, Systemic chemotherapy, Chronic lymphopenia or corticosteroids, ICI	8
Hashemi et al. (2020) [29]	USA	363	63.2±13.2	201 (55.37)	Multi-center retrospective study	Cardiac diseases	39 (10.7)	OR 0.98 (0.46-2.09)	Death	CLD, age, obesity, gender, HTN, diabetes, hyperlipidemia, pulmonary disorders	7
Lanza et al. (2020) [30]	Italy	222	66.4 (53.8–75.8)	163 (73)	Observational retrospective study,	Heart disease	27 (12.16)	OR 1.19 (0.58-2.44)	In-hospital death	Age, gender, smoke habit, CRP, Lung disease, cancer, diabetes, CKD, CURB-65a 1, CURB-65a 2, diabetes, BMI	8
Zeng et al. (2020) [31]	China	461	45.00 (34.50-57.00)	239 (51.84)	Multicenter retrospective study	CVD	25 (5.42)	HR 2.30 (0.99-5.38)	Severity	Age, gender, HTN, diabetes, hematology, biochemistry, infection-related indices, coagulation function	8
Petrilli et al. (2020) [32]	USA	5279	54 (38-66)	2615 (49.5)	Prospective cohort study	Coronary artery disease	704 (13.3)	OR 1.08 (0.81-1.44)	Mortality	Age, gender, BMI, race, COPD and asthma, diabetes, HTN, cirrhosis, CKD, CAD, immunosuppression, cancer, tobacco smoking	8
Arshad et al. (2020) [33]	USA	2541	63.7±16.5	1298 (51.1)	Retrospective cohort study	Cardiovascular Comorbidity	222 (8.7)	HR 1.062 (0.8-1.410)	Death	HCQ alone (vs. neither medication), azithromycin alone (vs. neither medication), HCQ+AZM (vs. neither medication), age, gender, ethic, BMI,lung comorbidity,CKI comorbidity,COPD, HTN,asthma, COPD,cancer,diabetes, percent O2 saturation < 95, admission to ICU, ventilator, given steroid, given tocilizumab	7
San Román et al. (2020) [34]	Spain	522	68±15	294 (56)	NR	Heart disease	68 (13.02)	OR 2.017 (1.050-3.876)	Severity	Age, SatO2 <90%, creatinine > 1.5 mg/dL, c-reactive protein> 10 mg/L	7
Cheng et al. (2020) [35]	China	456	54.97±18.59	211 (46.27)	Retrospective cohort study	CVD	52 (11.4)	OR 1.204 (0.554-2.619)	Any in-hospital disease progression	Age, gender, HTN, diabetes, CKD, neural system diseases, pulmonary disease, cancer, laboratory findings(leucocytes count, neutrophil count, lymphocyte count, NLR, platelet count, albumin, APTT, prothrombin time, INR, D-dimer, aspartate aminotransferase, creatinine, potassium, creatine kinase, lactate dehydrogenase, procalcitonin, C-reactive protein, erythrocyte sedimentation rate, IL-6	8
Oussalah et al. (2020) [36]	France	149	65 (54–77)	91 (61.1)	Retrospective, longitudinal cohort study	CVD	38 (25.5)	OR 2.35 (0.35-15.68)	Death	Age, COPD, gender, creatinine >10.1 mg/L, HTN	8
Kim et al. (2020)[37]	Korea	9148	51*	3556 (38.9)	Observational Study	Heart failure	124 (1.4)	OR 3.17 (1.88–5.34)	Mortality	Gender, age, type of distiricts, high epidemic region and socio-economic status	8
Chen et al. (2020) [38]	China	3309	62(49-69)	1642 (49.6)	Retrospective	CVD	242 (7.3)	OR 1.41 (0.94-2.13)	Death	Age, gender, HTN, diabetes, cerebrovascular disease, malignancy, CKI, COPD, days from onset to clinics (vs ≤5d), days from onset to admission (vs ≤12d)	9
Ferrante et al. (2020) [39]	Italy	332	66.9 (55.4-75.5)	237 (71.4)	Single-center cohort study	CAD	49 (14.5)	OR 2.14 (0.99-4.63)	Death	Age, HTN, CVA, Cancer, eGFR, PaO/FiO2 ratio, PA diameter, baseline ACEI/ARB use	7
Rastad et al. (2020) [40]	Iran	2597	54.8±16.9	1589 (53.7)	Retrospective cohort study	CVD	314 (10.6)	OR 0.61 (0.30, 1.24)	In-hospital mortality	WBC, neutrophils, lymphocytes, serum concentrations, creatinine, LDH, AST, ALT, Hb, ESR, CRP, age	8
Hwang et al. (2020) [41]	South Korea	103	67.62±15.32	52 (50)	Retrospective cohort study	CVD	12 (12)	HR 2.556 (0.535–12.207))	Mortality	Age, diabetes, CLD, Alzheimer’s dementia, stroke	7
Grasselli ei al. (2020) [42]	Italy	3988	63 (56-69)	3188 (79.9)	Retrospective, observational cohort study	Heart disease	533 (13.4)	HR 1.08 (0.91-1.29)	Death	Age, gender, respiratory support, HTN, hypercholesterolemia, type 2 diabetes, Malignancy, COPD, ACE inhibitor therapy, ARB therapy, statin, diuretic, PEEP at admission, FiO2 at admission, PaO2/FiO2 at admission	8
Deng et al. (2020) [43]	China	264	64.5 (53.3-74.0)	130 (49.2)	Retrospective study	Coronary heart disease	32 (12.1)	HR 1.855 (1.006-3.421)	Death	Age, gender, HTN, cTnI-ultra, CK-MB, MYO, NT-proBNP, Cr	7
AI-Salameh et al. (2020) [44]	France	433	72±14.3	226 (52.1)	Observational cohort	CVD	99 (31.2)	HR 1.84 (1.1-3.08)	Death	Age, diabetes, gender, abnormal LFTs	7
Atkins et al. (2020) [45]	UK	507	74.3±4.5	311 (61.3)	NR	CHD	108 (21.5)	OR 0.86 (0.55-1.36)	Death	Age, gender, race, education, atrial fibrillation, stroke, HTN, diabetes (type 2), CKD, depression, dementia, asthma, COPD, osteoporosis, osteoarthritis, delirium, pneumonia, falls/fragility fractures	8
Yao et al. (2020) [46]	USA	242	66.1±18.3	104 (42.98)	Single-institution retrospective study	Heart Disease	39 (13.6)	HR 0.94 (0.43-2.07)	Mortality	Zinc sulfate (yes vs no), age, gender, COPD, clinical severity, lopinavir/ritonavir, steroids, IL-6 receptor inhibitors,	8
Pinto et al. (2020) [47]	Italy	1226	71.7±14.5	733 (59.8)	Observational cohort Study	CVD	NR (NR)	OR 1.58 (0.68–3.68)	Death	Age, sex, presence of metastatic disease, time since cancer diagnosis	7
Chilimuri et al. (2020) [48]	USA	375	63.0 (52.0-72.0)	236 (63)	Retrospective cohort study	CVD	62 (17)	OR 1.56 (0.78-3.11)	Mortality	Age, gender, HTN, lymphocyte, creative protein, alanine aminotransferase, aspartate aminotransferase, creatine kinase	8
Lian et al. (2020) [49]	China	232	NR	108 (46.5)	Retrospective study	Heart disease	31 (13.36)	HR 2.587 (1.156-5.787)	Severity	Age, NLR, multiple mottling and ground-glass opacity	8
Zhao et al. (2020) [50]	USA	641	58.9±17.5	358 (55.85)	Retrospective study	Heart failure	20 (3.12)	OR 33.48 (4.99-224.45)	Mortality	LDH, procalcitonin, smoking history, SpO2, lymphocyte count, procalcitonin, LDH, COPD, SpO2, heart rate, age	8
Wang et al. (2020) [51]	USA	1827	52.7±21.1	500 (32.6)	NR	CVD	589 (32.2)	OR 2.21 (1.21-4.04)	Severity	Gender, race, marital status, Insurance type, smoking history, BMI, comorbidities (diabetes, COPD, CKD, CLD, HTN, allergic rhinitis), SABA, combination	7
Garcia-Azoin et al. (2020) [52]	Spain	576	67.18±14.75	326 (56.6)	Retrospective cohort study	Cardiac disease	154 (26.7)	OR 1.20 (0.730-1.999)	Mortality	mRS≥3, age, gender, HTN, diabetes, smoking, pulmonary disorders, cancer, chronic neurological disorders, immunosuppression	7
Alkhatib et al. (2020) [53]	USA	158	57±15.1	61 (38.6)	Retrospective cross-sectional analysis	Heart Failure	21 (13.3)	OR 2.4 (0.734-7.845)	Severity	Age, gender, diabetes, HTN, lung disease, CKD, BMI	7
Hernández-Galdamez et al. (2020) [54]	Mexico	211003	45.7±16.3	115442 (54.71)	Cross-sectional study	CVD	4949 (2.35)	OR 0.93 (0.87-1.00)	Death	At least one comorbidity/risk, CKD, immunosuppression, diabetes, COPD, HTN, asthma, obesity, smoking	8
Bellmann-Weiler et al. (2020) [55]	Australia	259	66.8±14.3	157 (60.62)	Retrospective	CVD	152 (58.62)	OR 2.127 (0.309–14.647)	Death	Age, CKD, COPD, eGFR, leukocytes, PCT, anemia,	8
Berenguer et al. (2020) [56]	Spain	4035	70 (56 – 80)	2433 (61)	Retrospective nationwide cohort study	Chronic heart disease	932 (23.3)	HR 1.58 (1.38-1.81)	Death	Gender, age, HTN, diabetes, COPD, obesity, CKI stage 4, liver cirrhosis, chronic neurological disorder, cancer, dementia, headache, myalgia/arthralgia, anosmia, cough, sputum production, dyspnea, chest pain, vomiting/nausea, altered consciousness, low SaO2, WBC count, neutrophil-to-lymphocyte ratio, platelets, prolonged APTT, eGFR, ALT, CRP	7
Gottlieb et al. (2020) [57]	USA	8673	41 (29 – 54)	4045 (46.6)	Retrospective case-control study t	Congestive Heart Failure	218 (14.7)	OR 1.45 (1.00-2.12)	Critical Illness	Age, gender, race, COPD, HTN, hyperlipidemia, diabetes, prior CVA, CKD, current ESRD, obstructive sleep apnea, bloodborne cancer, symptoms (anosmia, cough, headache, myalgias), labs(WBC, ALC,ANC/ALC, total Bilirubin, albumin, AST, ALT, LDH, lactate, D-Dimer, CRP, ferritin, troponin)	8
Agarwal et al. (2020) [58]	USA	1126	67.9±13.7	630 (49.3)	Retrospective	CVD	754 (59)	OR 1.18 (0.88-1.57)	Mortality	Treatment regimen (noninsulin only, insulin 1 noninsulin, insulin only), HTN, CKD, COPD	7
Shang et al. (2020) [59]	China	2529	66	73 (64.6)	Retrospective	CHD	28 (24.8)	OR 5.611 (1.392-22.623)	Death	Age, D-dimer, PCT, LYM, diabetes, CRP, BUN	8
Shi et al. (2020)[60]	Iran	386	59.46±15.82	236 (61.1)	Prospective, single-center study	CVD	97(25.1)	HR 1.121 (0.565-2.226)	Death	Age, diabetes, malignancy, CKD, CVA/TIA, previous ACEI/ARB use, ARDs, AKI	7
Posso et al. (2020) [62]	Spain	834	60	400 (46.5)	Retrospective	Heart Failure	37 (37.4)	OR 1.6 (1.01-2.55)	Death	Age, gender	7
Shu et al. (2020) [63]	China	571	50.0 (38.0-59.0)	278 (48.7)	Single-center, retrospective cohort study	Coronary heart disease	12 (2.1)	OR 6.75 (0.629-72.61)	Severity	Smoke, HTN, diabetes, dyspnea, consolidation, interstitial abnormalities, lymphocyte counting	8
Parra-Bracamonte et al. (2020) [64]	Mexico	142690	45 (34.0-57.0)	79280 (56)	NR	Cardiopathy	3521 (2.0)	OR 1.012 (0.92-1.112)	Mortality	Age, gender, smoking, hospitalized, pneumonia, comorbidity (HTN, obesity, diabetes, COPD, asthma, immunosuppressed, CKD, other complication)	8
Pablos et al. (2020) [65]	Spain	456	65±17.9	182 (41)	Retrospective observational matched cohort study	Heart failure	106 (23.2)	OR 1.57 (0.93-2.66)	Composite severe COVID-19 outcome	CTD, age, gender, obesity, diabetes, glucocorticoids (any dose), antivirals	8
Zhang et al. (2020) [66]	China	461	51 (38-64)	264 (57.3)	Multicenter study	Coronary heart disease	25 (5.4)	OR 0.382 (0.096-1.526)	Critical illness	Age, gender, comorbidities (HTN, diabetes, CLD), types of previous surgery (gastrointestinal surgery, urogenital surgery, skeletal surgery, cardiovascular surgery, others), WBC, neutrophil, lymphocyte, LDH, hemoglobin, platelet, albumin, AST, ALT, DBIL, IBIL, TBIL, APTT, PT, D-dimer, creatinine, hs-CRP, procalcitonin, urea nitrogen, FBG, CT score)	8
Fox et al. (2020) [67]	USA	389	66.2±14.2	208 (46.5)	Single-center retrospective analysis	CAD	77 (19.79)	OR 1.579 (0.562–4.436)	In-hospital mortality	Age, BMI, gender, ethnic, Hispanic, others, COPD, asthma, CAD, HTN, atrial fibrillation, CKD	7
Vena et al. (2020) [68]	Italy	317	71 (60-82)	213 (67.2)	Retrospective study	CVD	63 (19.9)	OR 2.58 (1.07-6.25)	All-cause in-hospital mortality	AKI, age, CRP, IL-6	7
Ng et al. (2020) [69]	USA	10482	66	6239 (59.5)	Retrospective study	Heart Failure	920 (8.78)	OR 1.32 (1.14-1.53)	Death	Age, sex, race/ethnicity, BMI, diabetes mellitus, HTN, cancer, mechanical ventilation, use of vasoactive medication, hemoglobin, lymphocyte, blood urea nitrogen, albumin, C-reactive protein and ferritin	8
He et al. (2020) [70]	China	288	48.5 (34.3-62)	131(45.5)	Single-center, retrospective cohort study	CVD	85 (29.5)	OR 0.986 (0.052-18.588)	Death	Age, CKD, exposure history in Wuhan >2 weeks, diarrhea, WBC count, lymphocyte count, creatinine, PCT,	8
Gupta et al. (2020) [71]	USA	2626	63.99±16.49	1497(57.00)	Retrospective study	CAD	516 (19.6)	OR 1.179 (0.844-1.647)	In-hospital mortality	Age, gender, CKD, exposure history in Wuhan >2 weeks, diarrhea, white blood cell count, lymphocyte count, creatinine	6
Czernichow et al. (2020) [72]	Europe	5795	59.8±13.6	3791 (65.4)	Prospective cohort study	HF	264 (4.55)	OR 1.15 (0.82-1.59)		Body mass index, age, diabetes, hypertension, dyslipidemia, sleep apnea, CKD, malignancies, history of smoking, gender	8
Sisó-Almirall et al. (2020) [73]	Spain	322	56.7±17.8	161 (50.0)	Multicenter, observational descriptive study	HF	25(7.8)	OR 1.92 [0.74–4.84]	Death or ICU admission	Age, gender	7
Brenner et al*. (2020) [74]	Germany	9548	62.1	4182 (43.8)	Ongoing statewide cohort study	CVD	4186 (43.8)	HR 1.285 (0.936–1.763)	Mortality	Any cause, age, gender, cancer, respiratory disease, Season	8
De Rossi et al. (2020) [75]	Italy	158	66.38±13.44	113 (71.52)	Retrospective cohort study	Heart disease	33 (20.89)	HR 3.001 (1.422-6.332)	Mortality	GROUP, age, gender, diabetes, HTN, CRP at admission, time to hospitalization, Time to hospitalization	7
Nimkar et al. (2020) [76]	USA	327	71 (59–82)	182 (55.7)	Retrospective case series	Cardiac Disease	98 (29.9)	OR 1.7 (0.7–3.9)	Mortality	AKI, ARDS, demographics (age, gender, race), HTN, diabetes mellitus, overweight (25 - 29.9), obese ( >= 30), underweight < 18.5	7
Klang et al. (2020) [77]	USA	1320	74.48±12.88	772 (58.48)	Multicenter observational retrospective study	CHD	258 (19.55)	OR 1.00 (0.8–1.4)	Death	Age, CAD, HTN, diabetes, CKD, COPD, cancer, obesity, smoking	7
Emami et al. (2021) [78]	Iran	1239	51.48±19.54	692 (55.9)	NR	CVD	132 (10.7)	HR 3.52 (1.23–11.15)	Mortality	Age, diabetes, chronic liver disease, cancer, HIV, smoking, asthma, immunodeficiency disease	5
Liu et al. (2020) [79]	China	2044	62.0 (51.0-70.0)	1000 (48.92)	Mini-national multicenter, retrospective, cohort study	CHD	199 (9.76)	OR 1.65 (1.02-2.66)	Critical disease (vs. moderate and severe disease)	Factors with effect modification, HTN, COPD, age, diabetes, tumor, CKD, cough	6
Giorgi et al. (2020) [61]	Italy	2653	63.2	1328 (50.1)	Population-based prospective cohort	CHD	168 (7.1)	HR 1.7 (1.2–2.5)	Death	Age, gender	7
Feng et al. (2020) [81]	China	114	63.96±13.41	71 (62.3)	Single-center, prospective study	CVD	31 (27.2)	HR 1.062 (0.380–2.970)	Poor outcome	Age, gender	7
Li et al. (2020) [82]	China	199	67 (61-78)	89 (44.7)	Retrospective study	CVD	NR (NR)	OR 0.250 (0.020-3.155)	Death	Age, CKD, HTN, Diabetes, d-dimer at admission, lymphocyte count at admission, fasting plasma glucose at admission, treatment with low molecular weight heparin, Antidiabetic drugs	7
Seiglie et al. (2020) [83]	USA	450	63.32±17.13	259 (57.5)	Observational study	CHF	52 (11.56)	OR 1.94 (0.78-4.85)	Death	Diabetes, BMI category (overweight, Obese), age, male, race/ethnicity (Hispanic, African American, other, unknown/missing), HTN, COPD/asthma, cancer (active), liver disease, renal disease	7
Tural Onur et al. (2020) [84]	Turkey	301	57±18	206 (68.4)	Retrospectively	CVD	19 (6.3)	OR 15.331 (3.394-69.272)	Death	Age, length of stay, lung cancer	7
Anzola et al. (2020) [85]	Italy	431	65±16	263 (61)	Prospective study	CVD	77 (18)	OR 0.618 (0.297-1.285)	Death	Age, lymphocyte count, creatinine, AST, CRP, diabetes, HTN, gender (male),	7
Ioannou et al. (2020) [86]	USA	10131	61.6±15.9	9221 (91.0)	Longitudinal cohort study	CAD	2203 (21.7)	HR 1.02 (0.88-1.18)	Death	Diabetes, cancer, HTN, congestive heart failure, cerebrovascular disease, dialysis, chronic kidney disease, cirrhosis, asthma, COPD, obstructive sleep apnea, obesity, hypoventilation, alcohol dependence, smoking, Charlson comorbidity body index score	9
Bahl et al. (2020) [87]	USA	1461	62.0 (50.0–74.0)	770 (52.7)	Multicentered cohort study	CVD	163 (11.2)	HR 1.32 (0.95–1.83)	Mortality	Age, gender, race (Black/African American, White/Caucasian, other), diabetes mellitus, HTN, respiratory rate, blood oxygen saturation White blood cell count, hemoglobin, ALT, creatinine, d-dimer, procalcitonin, lactic acid	6
Kabarriti et al. (2020) [88]	USA	5902	58 (44-71)	2768 (46.9)	Cohort study	CVD	1306 (22.1)	HR 1.20 (1.03-1.41)	Death	Age, gender, socioeconomic status (Lowest quartile, Second quartile, third quartile, highest quartile)	8
Jackson et al. (2020) [89]	USA	51	60 (45–69)	29 (56.9)	Retrospective observational cohort	CAD	10 (19.6)	OR 2.37 (1.08–5.23)	Death	End-stage renal disease, neurologic disorders,	6
Desai et al. (2020) [90]	Italy	575	64.8 (27-93)	380 (66.09)	Single-center, retrospective, observational study	CVD	155 (27.1)	HR 1.78 (1.21–2.61)	Death	Age, ACEi, therapy: LMWH	8
Wang et al. (2021) [91]	China	663	58 (44-69)	321 (48.4)	Retrospective	CVD	164 (24.7)	OR 1.66 (0.82-3.47)	Poor therapeutic effect	Age, gender, respiratory diseases, urinary diseases, T2DM, severe and critical condition, Fever, Expectoration, dyspnea, chest tightness, muscle aches, dizziness, neutrophil count >6.3 × 10 per L, Lymphocyte count <1.1 × 10 per L, Hemoglobin <115 g/L, ALT >40 U/L, ALT >40 U/L, Cr >73 mmol/L, Cr >73 mmol/L, albumin <35 g/L, LDH >300 U/L, CRP >10 mg/L	8
Solerte et al. (2020) [92]	Italy	169	69±1.0	115 (68)	Multicenter, case-control, retrospective, observational study	CVD	53 (38)	OR 2.5 (1.30–4.81)	Mortality	Treatment with sitagliptin, age, gender, cancer, chronic kidney disease, use of hydroxychloroquine use of antiviral agents	8
Hayek et al. (2020) [93]	USA	5019	60.42±14.86	3165 (63.06)	Multicenter cohort study	CAD	676 (13.47)	OR 1.13 (0.87-1.47)	In-hospital cardiac arrest	Number of intensive care unit beds ( ≥100 (reference), 50-99, <50), age, gender, Black compared with non-Hispanic white, Hispanic compared with non-Hispanic white, body mass index per 5 kg/m2,current or former tobacco use, diabetes mellitus, HTN, coronary artery disease, congestive heart failure, kidney disease (chronic or end stage), COPD, active malignancy, mSOFA score per 2 units	8
Chen et al. (2020) [94]	China	2828	60.0 (50.0-68.0)	1442 (51.0)	single-center Retrospective cohort study	CHD	181 (6.4)	OR 3.09 (1.69-5.64)	Adverse outcomes ( death, ARDS, respiratory failure and septic shock during hospitalization, mechanical ventilation, ICU admission, as well as clinical cure and discharges)	Age, COPD, AKI, Hs-CRP, neutrophil, lymphocyte, blood pressure	5
Lee et al. (2020) [95]	South Korea	5061	45.44±17.92	2,229 (44%)	Retrospective cohort study	CVD	49 (0.97)	HR 2.316 (1.053-5.094)	Mortality	Age, gender, cerebrovascular disease, HTN, diabetes, pulmonary disease, malignancy, CKD	8
Nachega et al. (2020) [96]	South Africa	766	46 (34–58)	500 (65.6)	Retrospective cohort study	Heart disease	30 (3.9)	HR 1.40 (0.68–2.88)	Death	Age, gender, clinical stage at admission (mild or moderate, Severe or critical, HTN, diabetes, obesity, asthma/chronic obstructive pulmonary, chronic kidney disease, cancer, HIV, current tuberculosis, chloroquine/azithromycin–based, received oxygen	8
Rozaliyani et al. (2020) [97]	India	4052	45.8±16.3	2169 (53.5)	Retrospective cohort study	Heart disease	148 (6.9)	OR 1.43 (0.85-2.41)	Death	Age, gender, registered address (West Jakarta, Central Jakarta, South Jakarta, East Jakarta, North Jakarta, outside Jakarta, citizenship, foreigner), Symptoms (cough, fever, malaise, dyspnea, headache, nausea/emesis, Sore throat, cold/runny nose, myalgia, chills, abdominal pain, diarrhea, pneumonia), temperature, comorbidity (HTN, COPD, diabetes, renal disease, malignancy, immunological disorder, liver failure, Obesity)	7
Wang et al. (2020) [98]	China	293	59.2 (42.8-73.1)	138 (47.1)	Retrospective study	Coronary heart disease	21 (7.2)	HR 1.771 (1.013-3.097)	Mortality	Age, gender, fever, cough, expectoration, dyspnea, catarrhal symptoms, neuromuscular symptoms, digestive symptoms, comorbidity, Hypertension, diabetes, cerebrovascular disease, COPD, chronic renal disease, chronic liver disease, malignancy, only one comorbidity, ≥2 comorbidities, complications, shock, acute cardiac injury, acute renal injury, acute liver injury, Only one complication, ≥2 complications	8
Liu et al. (2020) [99]	China	77	63.6±3.6	48 (62)	Retrospective study	CVD	15 (20)	HR 2.533 (1.108-6.306)	In-hospital death	HbA1C, age, gender, CRD	8
Al Kuwari et al. (2020) [100]	Qatar	5685	35.8±12.0	5052 (88.9)	Case series	CVD	250 (4.4)	OR 0.54 (0.24-1.22)	Severe or critical illness	Age, gender, Qatari nationality, HTN, diabetes mellitus, chronic lung disease, chronic kidney disease, cancer	8
Balbi et al. (2020) [101]	Italy	340	68 (57–76)	252 (74)	Retrospective observational study	CVD	86 (25)	OR 3.21 (1.28–8.39)	Death	Age, SpO2, PaO2/FiO2 ratio, Brixia score	6
Calmes et al. (2021) [102]	Belgium	493	58 ± 19	244 (49.49)	NR	Cardiopathy	88 (18)	OR 0.94 (0.53-1.7)	Intensive care unit stay	Age, gender	8
Talavera et al. (2020) [103]	Spain	576	67.18±14.75	325 (56.6)	Retrospective cohort study	Cardiological disorders	154 (26.7)	OR 1.201 (0.716-2.016)	Mortality	Age, sex, hypertension, diabetes, smoking habit, cardiological disorders, pulmonary disorders, cancer, and chronic neurological disorders	6
Zinellu et al. (2020) [104]	Italy	105	72.0 (59.5-80.0)	70 (66.67)	Retrospective	CVD	59 (56.19)	HR 2.53 (0.80-7.99)	In-hospital mortality	Age, gender, smoking status, intensity of care, respiratory disease, kidney disease, diabetes, cancer, De Ritis index ≥ 1.63	7
Mallow et al. (2020) [105]	USA	21676	64.9±17.2	11442 (52.8)	Retrospective cohort study	Severe heart disease	12000 (55.4)	OR 1.27 (1.16-1.40)	Mortality	Age, gender, insurance (Medicaid as any payer), teaching status (nonteaching hospital vs teaching hospital), hospital bed Size, chronic lung disease, moderate to severe asthma, immunocompromised, obesity, diabetes, CKD with dialysis, liver disease, HTN, DNR, statin use in hospital	8
Abbasi et al. (2020) [106]	Iran	262	58 (43–67)	172 (65.6)	Retrospective cohort study	CAD	78 (29.8)	OR 6.7 (1.08–42.2)	Mortality	Age, HTN, diabetes, chronic renal failure, hypoxia at admission, WBC, LYM count, LYM% less than 20%, Hb, Plt, AST, ALT, LDH, CRP, ESR, Cr, CT severity score	6
Craig-Schapiro et al. (2021) [107]	USA	136	56.24±35.04	93 (68.38)	NR	CVD	52 (38.23)	OR 0.76 (0.26-2.23)	Mortality	Waitlist status, age, gender, BMI, black, diabetes, pulmonary disease, history of stroke, smoking history, ACE / ARB use	7
Ryan et al. (2020) [108]	USA	556	57±17	296 (53)	Retrospective case-control study	CVD	71 (13)	OR 1.41 (0.77–2.58)	Composite of ICU Admission, Mechanical Ventilation, and Death	Age, immunocompromised status, dyspnea, vomiting, chronic kidney disease, COPD, diabetes mellitus, ACE inhibitor, gender, obesity, current or former smoker, obstructive sleep apnea, HTN, hyperlipidemia	6
Serin et al. (2020) [109]	Turkey	2217	47.66±17.23	1175 (53)	NR	CAD	165 (7.4)	HR 1.726 (0.645−4.618)	Mortality	COPD, chronic heart failure, HTN, diabetes mellitus, chronic renal failure, malignancy, without Involvement in CT, unilaterally, bilaterally, WBC, neutrophil, hemoglobin, C-Reactive Protein, D-Dimer, urea, aspartate aminotransferase, Lactate Dehydrogenase/Lymphocyte	5
Cao et al. (2020) [110]	China	101	56.6±15.1	67 (66.3)	Retrospective, two-center study	CVD	21 (20.8)	OR 0.439 (0.081–2.387)	Mortality	Age, respiratory rate, dyspnea, acute respiratory distress syndrome, diabetes, HTN, chronic pulmonary disease, bacterial infection	7
Gupta et al. (2020) [111]	USA	3099	62 (51–71)	2003 (64.6)	Multicenter cohort study	CAD	390 (12.6)	OR 1.17 (0.65-2.13)	28-day mortality	Age, gender, Non–white race, HTN, diabetes mellitus, BMI, chronic kidney disease, congestive heart failure, active malignancy, ≤3 days from hospital to ICU admission, lymphocyte count <1,000 mm3, PaO2:FiO2, altered mental status, ICU Day 1, secondary Infection, ICU Day 1, vasopressors, coagulation Component of SOFA Score, Liver component of SOFA Score, urine output (ml/day), initial RRT modality initial RRT modality, hospital size (no. pre-COVID ICU beds), regional density of COVID-19 (quartiles)	7
Raparelli et al. (2021) [112]	Italy	3517	77.64±11.51	2346 (66.7)	Retrospective analysis	Congestive Heart Failure	539 (15.7)	OR 0.75 (0.56-1.00)	Death	AGE, IHD, T2DM, dementia, COPD, CLD, CKD, AD, fever, SOB, cough, admission in ICU, AKI, acute cardiac injury, shock, antivirals, tocilizumab, length of stay	6
Chinnadurai et al. (2020) [113]	UK	215	74 (60–82)	133 (61.9)	Single-center observational study	CVD	93 (43.3)	OR 1.20 (0.61–2.40)	Mortality	Age, care home resident, frailty, smoking, respiratory diseases	6
Rajter et al. (2020) [114]	USA	280	59.6±15.9	153 (64.6)	NR	Cardiac Disease	43 (15.4)	OR 1.51 (0.43-5.22)	Mortality	Treatment group (Ivermectin VS Control), age, gender, current or former smoker, Race (Black, Hispanic, Other, White), comorbidities (diabetes, pulmonary, HTBN, No comorbidities), BMI, severe presentation, Intubated at study entry, MAP < 70 mm Hg, corticosteroid treatment, peripheral white cell count, lymphocyte count	7
Naaraayan ey al. (2020) [115]	USA	362	71 (59–82)	200 (55.3)	Retrospective case series	Cardiac diseases	119 (32.9)	OR 0.9 (0.5–1.4)	In-hospital mortality	age, sex, hypertension, diabetes, race, chronic obstructive pulmonary disease, renal disease and obesity	6
Cherri et al. (2020) [116]	Italy	53	75 (68–83)	32 (60.4)	Retrospective study	Cardiopathy	20 (37.7)	OR 1.15 (0.187-7.13)	Mortality	Age, BMI, diabetes, active oncological disease	7
Rodríguez-Molinero et al. (2020) [117]	Spain	418	65.4±16.6	238 (56.9)	Observational cohort study	Heart failure	26 (6.22)	OR 1.16 (0.44–3.06)	Case fatality	Age, gender, diabetes mellitus, obesity, chronic kidney disease, HTN, atrial fibrillation, dementia, OSAS, Auto-immune disease	6
Clift et al. (2020) [118]	UK	8256158	44.33±27.42	4111197 (49.8)	Cohort study	Heart failure	96225 (1.17)	HR 1.14 (1.08–1.20)	Death	No learning disability, learning disability apart from down syndrome, down syndrome, males vs. females, Townsend material deprivation score (5-unit increase), White, Indian British, Pakistani British, Bangladeshi British, Other Asian British, Caribbean British, Black British, Chinese British, other ethnic group, not in care home or homeless, lives in residential or nursing home, Homeless according to GP records, No kidney failure, chronic kidney disease stage, chemotherapy grad, blood cancer, bone marrow or stem cell transplant in past 6 month, respiratory tract cancer, Radiotherapy in past 6 month, Solid organ transplant (excluding kidney and bone marrow), immunosuppressant drug, ≥4 scripts from GP in past 6 mo, Leukotriene or LABA, ≥4 scripts in past 6 month, Oral steroids, ≥4 scripts in past 6 month, Sickle celI disease or severe immunodeficiency, type 1 diabetes, type 2 diabetes, COPD, asthma, rare lung conditions (bronchiectasis, CF, or alveolitis), pulmonary hypertension or pulmonary fibrosis, coronary heart disease, stroke, atrial fibrillation, congestive heart failure, thromboembolism, peripheral vascular disease, congenital heart disease, dementia, Parkinson disease, Epilepsy MND, MS, myasthenia gravis, or Huntington disease, Cerebral palsy, severe mental illness, osteoporotic fracture (hip, spine, wrist, or humerus), rheumatoid arthritis or SLECirrhosis	9
Clift et al. (2020) [119]	UK	6083102	48.21±18.57	3035409 (49.90)	Population based cohort study	Coronary heart disease	215069 (3.54)	HR 1.24 (1.10-1.40)	Death	No learning disability, learning disability apart from down syndrome, down syndrome, males vs. females, Townsend material deprivation score (5-unit increase), White, Indian British, Pakistani British, Bangladeshi British, Other Asian British, Caribbean British, Black British, Chinese British, Other ethnic group, not in care home or homeless, lives in residential or nursing home, homeless according to GP records, No kidney failure, chronic kidney disease stage, chemotherapy grad, blood cancer, bone marrow or stem cell transplant in past 6 month, respiratory tract cancer, radiotherapy in past 6 month, solid organ transplant (excluding kidney and bone marrow), immunosuppressant drug, ≥4 scripts from GP in past 6 month, Leukotriene or LABA, ≥4 scripts in past 6 month, Oral steroids, ≥4 scripts in past 6 month, sickle celI disease or severe immunodeficiency, type 1 diabetes, type 2 diabetes, COPD, asthma, rare lung conditions (bronchiectasis, CF, or alveolitis), pulmonary hypertension or pulmonary fibrosis, coronary heart disease, stroke, atrial fibrillation, congestive heart failure, thromboembolism, peripheral vascular disease, congenital heart disease, dementia, Parkinson disease, epilepsy MND, MS, myasthenia gravis, or Huntington disease, cerebral palsy, severe mental illness, osteoporotic fracture (hip, spine, wrist, or humerus), rheumatoid arthritis or SLEcirrhosis	9
Gamberini et al. (2020) [120]	Italy	2540	66 (59–72)	300 (76.7)	Multicenter prospective observational study	Chronic ischemic heart disease	35 (9)	HR 0.277 (0.181–0.423)	Mechanical ventilation	Age, SOFA score at ICU admission, renal replacement therapy during ICU stays, lowest PaO2/FiO2 within 5 days, CRS < 40 mL/cmH2O within 5 days, neurologic complications	7
Omrani et al. (2020) [121]	Qatar	1409	39.82±14.2	1167 (82.8)	Retrospective cohort study	Coronary artery disease	31 (2.4)	OR 1.090 (0.449–2.643)	Admission to ICU	Age, gender, diabetes mellitus, HTN, chronic liver disease, chronic kidney disease, BMI	6
Yahyavi et al. (2020) [122]	Iran	2553	58.1±17.9	1498 (58.7)	Retrospective cohort study	CVD	942 (36.9)	OR 1.1 (0.8-1.5)	Mortality	angiotensin-converting enzyme inhibitors, angiotensin receptor blockers, chronic kidney disease, chronic pulmonary disease, diabetes mellitus, intensive care unit, diuretics, beta-blockers, and calcium channel blockers	7
Guisado-Vasco et al. (2020) [135]	Spain	607	69±22.0	394 (65.02)	Retrospective, observational, longitudinal study	Chronic cardiac disease	133 (22.62)	OR 1.956 (0.778-4.922)	In-hospital death	Age, gender, Chest X-ray score, hydroxychloroquine, tocilizumab, lopinavir/ritonavir, cyclosporine A, Glucocorticoids, Lymphocyte count at admission, Ferritin at admission, C-reactive protein at admission, lactate dehydrogenase (LDH) at admission, d-dimer at admission, Creatinine at admission, arterial hypertension, diabetes mellitus, chronic respiratory disease, PaO2/FiO2	7
Izzy et al.* (2020) [124]	USA	5190	52 (36–66)	2378 (46)	NR	Coronary artery disease	257 (5)	OR 0.52 (0.323–0.835)	ICU Admission	Age, gender, smoking status, last BMI, comorbidities (diabetes mellitus, hyperlipidemia, HTN, obstructive lung disease, interstitial lung disease, cerebrovascular disease, obstructive sleep apnea, CKD, transplantation, auto-immune diseases, malignancy), total comorbidities (0, 1–2, >2)	8
Chow et al. (2020) [125]	USA	412	55 (41-66)	244(52.9)	Retrospective, observational cohort study	CAD	52 (12.62)	HR 1.91 (1.06-3.42)	In-hospital death	Age, gender, BMI, Ethnicity (African American, Asian, Hispanic/Latino), HTN, DM, renal disease, aspirin use	6
Raines et al. (2020) [126]	USA	440	60.8±14.07	393 (89.32)	Retrospective	CVD	364 (82.73)	OR 0.9 (0.47-1.73)	Mortality	Age, gender, race, BMI, immunodeficiency syndromes, pulmonary diseases, oncologic diseases, gastrointestinal diseases, renal diseases, hematologic diseases, endocrine diseases, neurologic problems, lifetime tobacco user	7
Ramos-Rincon et al. (2020) [123]	Spain	2772	86.3 (83.2-89.6)	1367 (49.4)	Nationwide, multicenter, retrospective, observational study	CVD	855 (30.8)	OR 1.22 (0.96-1.54)	Mortality	Age, gender, degree of dependence (independent or mild, moderate, Severe), comorbidities ( Charlson comorbidity Index, non-atherosclerotic cardiovascular disease, atherosclerotic cardiovascular diseases, dementia, obesity, moderate-severe renal disease), symptoms (shortness of breath, anorexia, diarrhea), physical exam (Oxygen saturation < 90% (pulsi oximetry), temperature 37.8 ºC, HTN (systolic blood pressure<100 mmHg), tachycardia (>100 beats per minute), Tachypnoea (20 breaths per minute), confusion, pulmonary rales, qSOFA score 2 (high risk)), chest X-ray (normal, unilateral infiltrates, bilateral infiltrates), laboratory findings (leukocytes 10.0 x103/L, neutrophils 7.5 x103/L, Lymphocytes<0.800 x103/L, monocytes<0.500 x103/L, pH<7.40, PO2, PO2/FiO2 ratio < 200, glucose > 126 mg/dL, eGFR < 45ml/min/1.73m2, lactate dehydrogenase 500 U/L, AST,ALT, CRP, venous lactate, procalcitonin, interleukin-6, d-dimer, serum ferritin)	6
Zhang et al. (2021) [127]	China	222	51.5 (34.0-65.3)	90(40.54)	NR	Chronic cardiovascular disease	44 (19.82)	HR 3.616 (1.111-11.776)	Mortality	Dyspnea, pharyngalgia, COPD, elevated myocardial enzymes, acute liver dysfunction, acute kidney injury	6
de Souza et al. (2020) [128]	Brazil	9807	70.21±8.37	4662 (47.5)	Retrospective population-based study	CVD	1192 (12.2)	OR 1.15 (0.95–1.39)	Mortality	Age, gender, initial symptoms reported (initial symptoms reported, fever, fatigue, headache, myalgia, odynophagia, dyspnea, diarrhea), comorbidities (diabetes, HTN, chronic lung disease, chronic kidney disease, obesity)	8
Kolhe et al. (2020) [129]	UK	1161	72.1±16.0	657 (56.59)	Retrospective cohort study	Congestive cardiac failure	207 (17.83)	OR 1.38 (0.95-1.99)	Mortality	Age, gender, ethnicity (White, Asian, Black, mixed, others, not stated), cerebrovascular disease, Dementia, chronic lung disease, connective tissue disorder, Diabetes with complication, paraplegia, chronic kidney disease, chronic liver disease, Cancer, treatment (ACEI or ARB use, ACEI or ARB use), AKI	8
Kim et al. (2021) [130]	USA	10861	65 (54-77)	6468(59.6)	NR	CAD	1447 (13.3)	OR 1.02 (0.90-1.17)	Death	Age, gender, race/ethnicity, BMI, HTN, DM,CKD, end stage renal disease, cancer, asthma, COPD, smoking status, hospital type	6
Giustino et al. (2020) [131]	New York City & Milan	305	63 (53–73)	205 (67.2)	International, multicenter cohort study	Heart failure	24 (7.9)	OR 5.38 (1.65-17.54)	In-Hospital Death	Age, Hispanic ethnicity, history of heart failure, cardiocirculatory shock, acute respiratory distress syndrome, acute kidney injury stage II or III, no cardiac injury (No cardiac injury vs cardiac injury with echocardiographic abnormalities)	7
An et al. (2020) [132]	Korea	228	44.97±19.79	107 (46.9)	Cohort study	CVD	70 (30.7)	HR 1.23 (0.89-1.70)	Mortality	Age, gender, income level, residence, household type, disability, symptom, infection route, underlying medical condition (none, HTN, diabetes mellitus, hyperlipidemia, cerebrovascular disease, cancer, chronic lung disease or asthma, chronic renal disease, mental illness, chronic liver disease)	6
Piazza et al. (2020) [133]	USA	1114	50.6±18.3	511 (45.9)	Retrospective observational cohort analysis	CAD	90 (8.1)	OR 1.09 (0.38–3.16)	Death	Major arterial or venous thromboembolic event (Age, gender, VTE prophylaxis, ARDS, d-dimer (decile))	7
Rao et al. (2020) [134]	China	240	48 (23–87)	111 (46.250	Retrospective cohort study	CVD	43 (17.9)	OR 3.326 (0.721-15.336)	Severe pneumonia	Age	7
Tehrani et al. (2021) [136]	Sweden.	255	66±17	150 (59)	Retrospective analysis	Chronic heart failure	34 (13)	OR 1.01 (0.42-2.42)	Death	Age, HTN, chronic kidney disease, previous stroke	8
Hyman et al. (2020) [137]	USA	755	63±13	483 (64.0)	Retrospective cohort study	Congestive heart failure or valve disorder	30 (4.3)	HR 1.39 (0.87–2.23)	Mortality	Hospital site, baseline demography, preexisting comorbidities, laboratory findings at admission, maximum vital sign values	7
Hamilton et al. (2020) [138]	UK	1032	71 (56–83)	569 (55.1)	Retrospective review	Congestive Heart Failure	129 (12.5)	HR 2.01 (1.51-2.67)	Mortality	AKI, cancer, other ethnicity, diabetes, gender, RAASi, race, dementia, myocardial infarction, age	6
Liu et al. (2020) [139]	China	774	64 (54–73)	452 (58.4)	Multicenter retrospective observational study	Chronic cardiac disease	91 (11.8)	HR 1.12 (0.68–1.84)	Mortality	Time-varying exposure, age, gender, APACHE II score, COPD, diabetes, HTN, chronic kidney disease, chronic liver disease, stroke, malignancy, immunosuppression, fever at admission, systolic pressure at admission, leukocytes, hemoglobin, platelets, lymphocytes, d-dimer, total bilirubin, serum creatinine, procalcitonin, corticosteroids, corticosteroids, human immunoglobulin	8
Ganatra et al. (2020) [140]	USA	2467	59 (18–101)	1032 (42)	Retrospective study	CAD	184 (7.0)	OR 0.92 (0.66–1.27)	Severe disease	Age, prior/current smoker, β-blockers, history of cancer, gender, diabetes mellitus, ACEi or ARB, HTN, COPD, CKD	4
Rubio-Rivas et al. (2020) [141]	Spain	12066	68 (56–79)	7052 (58.5)	Cohort study	Chronic heart failure	809 (6.7)	OR 1.16 (1.02–1.32)	In-hospital mortality	Age, gender, BMI, clusters, comorbidity (Arterial hypertension, diabetes mellitus, hyperlipidemia, hyperlipidemia, chronic kidney disease, chronic hepatopathy, active cancer), Charlson’s index, heart rate upon admission, respiratory rate upon admission > 20 bpm, PaO2/FiO2 upon admission, lab test upon admission (CRP mg/L, LDH U/L), treatments during admission (Redeliver, tocilizumab, corticosteroids)	9
Mendes et al. (2020) [142]	Switzerland	235	86.3±6.5	102 (43.4)	Retrospective monocentric cohort study	Heart failure	66 (28.1)	OR 1.51 (0.95-2.40)	Mortality	Gender	6
Nemer et al. (2020) [143]	USA	350	64±16	194 (55)	Prospective	Congestive heart failure	42 (12)	OR 0.76 (0.17-3.39)	Primary composite outcome was defined as death, ICU transfer, or increased oxygen requirement.	Age, BMI, COPD, peripheral oxygen saturation on room air, CRP, lactate dehydrogenase level, abnormal troponin T level, abnormal d-dimer level, Abnormal chest x-ray findings	8
Guo et al. (2020) [144]	China	350	43(32–56)	173(49.4)	Retrospective, multicenter study	CVD	15 (4.3)	OR 1.81 (0.42–7.84)	Severe COVID-19	Age, gender, Wuhan exposure, family cluster case, smoking, comorbidity (HTN, diabetes, chronic kidney disease, chronic liver disease, cerebral infarction)	6
Hilbrands et al. (2020) [145]	Netherlands	305	60±13	189(62)	Observational study	Heart failure	64 (21)	OR 1.39 (1.02–1.89)	28-day case-fatality	Age, gender	5
Wang et al. (2020) [146]	China	7283	64 (53–71)	3732 (51.2)	Retrospective observational study	CVD	161 (2.2)	HR 1.83 (1.33-2.51)	Death	Age, gender, location (central area in Wuhan, Other areas), occupation (medical workers, retirees, others), diabetes, HTN, respiratory disease, number of symptoms at admission, date of onset (Dec 2019–9 Jan 2020, 10–22 Jan 2020, 23 Jan–1 Feb 2020, 2–25 Feb 2020)	9
Tang et al. (2020) [147]	USA	752	73.9 (21.9-105.4)	323 (43)	Cohort study	Coronary heart disease	240 (31.91)	HR 0.83 (0.58-1.19)	Death	Age, gender, race, and facility	8
Annweiler et al. (2020) [173]	France	77	88 (85−92)	39 (50.6)	Retrospective quasi-experimental study	Cardiomyopathy	42 (54.5)	HR 4.04 (0.81-20.30)	14-day mortality	Age, gender, Iso resource groups score, severe undernutrition, history of cancer, history of HTN, glycated hemoglobin, number of acute health issue, use antibiotics, use of systemic corticosteroids, use treatments of respiratory disorder	5
Huang et al. (2020) [148]	China	676	56.0 (39.0–68.0)	314 (46.4)	Retrospective study	Heart Disease	71 (10.5)	HR 1.40 (0.76–2.47)	Hospital mortality	Age, gender, HTN, Diabetes, cancer, d-dimer, CRP, PCT, LDH	6
Poterucha et al. (2021) [149]	USA	887	64.1	513 (58)	Retrospective study	CAD	104 (12.0)	HR 1.56 (1.04-2.33)	Mortality	AF/AFL, QRS abnormality, ST-T wave abnormality, Initial hs-cTnT ≥ 20 ng/L, age, gender, Hypertension, Diabetes, CKD, primary lung disease, Obesity, HFrEF, HFpEF, active cancer, history of cancer	6
Li et al. (2020) [150]	China	100	62.0 (51.0–70.8)	56 (56.0)	NR	CVD	15 (15.0)	HR 3.73 (0.41–33.84)	Cardiac damage	Age, gender, Hypertension, diabetes, hyperlipidemia, white blood count, prothrombin time, d-dimer, creatinine interleukin-6, procalcitonin, hs-CRP	6
Prado-Galbarro et al. (2020) [151]	Mexico	9487	31.37 (41.13-51.18)	5050 (53.2)	Observational study	CVD	171(1.8)	HR 0.85 (0.67-1.06)	Mortality	Age, gender, indigenous ethnicity, pneumonia, COPD, diseases associated with immunosuppression, additional comorbidity (Chronic diseases interaction, HTN, diabetes, obesity, chronic kidney disease, intensive care unit), region, density, mode of transport (driving, public transport, walking)	8
Shah et al. (2020) [152]	USA	487	68.53±16.66	273 (56.06)	Retrospective review	Cardiomyopathy	16 (3.28)	OR 3.33 (1.07-10.41)	Mortality	Age, gender, patient admitted from home, PMH HTN, PMH hyperlipidemia, PMH A. fib, , PMH CVA, PMH diabetes, PMH dementia, PMH active cancer, AKI, Dyspnea in ED noted as positive, initial CXR/CT findings	7
Botta et al. (2021) [153]	Netherlands	553	67.0 (59.0–73.0)	417 (75)	National, multicenter, observational cohort study	Heart failure	25 (5.0)	OR 0.73 (0.26-2.08)	28-day mortality	Ventilatory variables on day 0 (positive end-expiratory pressure, tidal volume, respiratory system compliance), PaO2/FiO2, laboratory tests on day 0* pH, Lactate, Creatinine), vital signs on day 0 (Heart rate, mean arterial pressure), organ support on day 0 (use of vasopressor, fluid balance), demographic characteristics (age, gender, BMI, HTN, diabetes, chronic kidney disease, COPD, use of angiotensin-converting enzyme inhibitor, use of angiotensin II receptor blocker)	6
Di Domenico et al. (2020) [154]	France	310	64 (52–76)	200 (64.5)	Single‑center retrospective study	Heart disease	50 (16.2)	HR 1.921 (0.893-4.135)	Death	Age, diabetes, HTN, CKD, obesity, vascular disease, ever been a smoker	7
Ayaz et al. (2020) [155]	Pakistan	66	50.6±19.1	40 (61)	Retrospective cohort study	Ischemic heart disease	10 (15)	OR 26.5 (4.7–147.8)	Mortality	Age, diabetes, HTN, ICU admission, mechanical ventilation, bilateral infiltrates on chest radiography, neutrophil to lymphocyte ratio ≥3.3, INR ≥1.2	6
Hippisley-Cox et al. (2020) [156]	UK	8275949	48.47±18.41	4115973 (49.73)	Prospective cohort study	CVD	433631 (5.24)	HR 0.85 (0.66-1.10)	Admission to ICU	ACE inhibitor, angiobrnsin enzyme blocker, gender, material deprivation, ethnicity, geographical region, smoking status, BMI, chronic renal disease, atrial fibrillation, type 1 diabetes, type 2 diabetes, hypertension, asthma, COPD, Beta-blockers, calcium channel blockers, other diabetes drugs, sulfonylureas, biguanides, anticoagulants, antiplatelets, statins, statins, potassium-sparing diuretics	9
Tomasoni et al. (2020) [157]	Italy	692	66.5±13.3	415 (68.9)	Multicenter study	CAD	148 (21.4)	HR 1.20 (0.67-2.14)	In-hospital mortality	Age, gender, smoker, HTN, hyper dyslipidemia, Diabetes, atrial fibrillation, COPD, CKD, Treatment before hospitalization (ACE-i/ARBs/ARNI, mineralocorticoids, Beta-blockers, direct oral anticoagulants, warfarin, Statins), baseline findings (heart rate, Oxygen saturation), laboratory measurements (PaO2/FiO2, red blood cell count, hemoglobin, hematocrit, lymphocytes count, platelets count, creatinine, eGFR (CKD-EPI), CRP on admission, procalcitonin, troponin, NT-proBNP, d-dimer, aspartate transaminase, albumin, international normalized Ratio)	7
Elmunzer et al. (2020) [158]	North American	1846	59.9±16.4	1044 (56.6)	Large-scale retrospective cohort study	Congestive Heart Failure	284 (15.4)	OR 1.60 (1.12-2.28)	Death	H2RA Use, PPI Use, age, gender, race, dementia, number of comorbidities, WBC at admission, platelets at admission, AST at admission, albumin at admission	6
Polverino et al. (2020) [159]	Italy	3179		2171 (68.3)	Nationwide observational study	Coronary artery disease	359 (11.3)	OR 1.11 (0.83-1.49)	Death	Age, gender, atrial fibrillation, blood cancer, COPD chronic renal failure, diabetes, HTN, obesity, organ cancer, stroke	5
Sharp et al. (2020) [160]	USA	21280	50 (34-66)	9053 (42.5)	Retrospective cohort study	Congestive Heart Failure	NA (NA)	OR 1.45 (1.18–1.77)	Adverse outcomes (death, ARDS, respiratory failure and septic shock during hospitalization, mechanical ventilation, ICU admission, as well as clinical cure and discharges)	Age, gender, BMI, coagulopathy, diabetes, fluid and electrolyte disorders, other neurological disorders, weight Loss, heart rate, systolic BP, oxygen saturation, respiratory rate	8
Stebbing et al. (2020) [161]	Italy&Spain	166	74.05±13.06	85 (51.2)	Observational studies	CVD	48 (28.9)	HR 1.41 (0.68-2.92)	Death & admission to ICU	Age, gender, HTN, diabetes, chronic Obstructive Lung disease, cronic kidney disease, Solid cancer, Charlson Comorbidity Index, baseline PaO2/FiO2, lymphocyte count (/mcL), alanine aminotransferase, hydroxychloroquine, lopinavir/ritonavir, glucocorticoids, low molecular weight heparin, antibiotics	6
Fu et al. (2020) [162]	China	355	43.5*	193 (54.37)	Hospital-Based Retrospective Cohort Study	Heart disease	20 (6.2)	OR 0.454 (0.102-2.010)	Myocardial injury	Age, gender, HTN, diabetes	7
Sheshah et al. (2020) [163]	Saudi Arabia	300	49.7±13.2	259 (86.3)	Single-center, retrospective study	Coronary Artery Disease	10 (3.3)	OR 19.4 (1.5-260)	Mortality	Age, gender, HTN, type 2 diabetes mellitus, chronic kidney disease, acute kidney injury, stroke, methylprednisolone, dexamethasone, hydroxychloroquine, azithromycin	6
Bowe et al. (2020) [164]	USA	5216	70 (61–76)	4908 (94)	Cohort study	CVD	1588 (30.0)	OR 0.87 (0.76-1.01)	Severe AKI	Age, gender, race, Smoking status, HTN, diabetes mellitus type 2, ACEI/ARB, diuretics, anticoagulant, immunosuppressants, b-blocker, aspirin, eGFR category	8
Cheng et al. (2020) [165]	China	220	59.5 (48.3-70.0)	106 (48.2)	Retrospective, observational study	CAD	22 (10.0)	HR 0.97 (0.35-2.68)	In-hospital death	Hypertension, history of cerebrovascular disease, History of diabetes mellitus, history of diabetes mellitus	4
Neumann-Podczaska et al. (2020) [166]	Poland	50	74.8±9.4	35 (70.0)	Retrospective	Heart disease	26 (52.0)	HR 2.61 (0.92–7.39)	60-day mortality	Age, functional Capacity, Diabetes	6
Ken-Dror et al. (2020) [167]	UK	429	70±18	242 (56.4)	Prospective cohort study	Chronic cardiac disease/congenital heart disease	103 (31.3)	OR 3.43 (2.1-5.63)	Mortality	Self-reported feverishness 38°C, cough self-report, oxygen saturation, history of fever, cough, sore throat, chest pain, muscle aches myalgia, altered consciousness confusion, obesity as defined by clinical staff, diabetes with complications, dementia, malnutrition, current admission to ICU/IMC/HDU, non-invasive ventilation BIPAP/CPAP, invasive ventilation, high flow nasal canula oxygen therapy, clinical pneumonia, inotropes vasopressors, viral pneumonia, bacterial pneumonia, anemia	7
Iannelli et al. (2020) [168]	France	8286	59.1±12.6	4296 (51.8)	Retrospective	Cardiac failure	569 (6.9)	OR 1.53 (1.24–1.89)	Death	Age, gender, cancer, diabetes, bariatric surgery	9
Sharifpour et al. (2020) [169]	USA	268	63±15	149 (55.6)	Cohort analysis	CAD	36 (13.4)	OR 1.381 (0.498–3.826)	Mortality	Age, CRP Slope d1to7, CRP tests (count d1 to 7), CRRT, CRP Max d1to7, obesity (BMI> = 30kg/m2), intubation, SOFA score, HTN	6
Martins-Filho et al. (2020) [170]	Northeast Brazil	1207	60 (46–73)	724 (60)	Retrospective cohort study	Heart failure	102 (8.45)	OR 2.00 (1.31–3.04)	Mortality	Infectious disease, kidney disease, age	6
Lee et al. (2020) [171]	Korea	7339	47.1±19.0	2970 (40.1)	Nationwide Population-Based Retrospective Study	CVD	455 (6.1)	OR 0.95 (0.64–1.40)	Death	Influenza, tuberculosis, COPD, pneumonia, asthma, DM, CKD, Chronic liver disease, HTN, malignancies, HIV infection, lopinavir/ritonavir, Hydroxychloroquine, ribavirin, type I interferon, Human immunoglobulin G, Oseltamivir, antibiotics, age, gender	8
Loffi et al. (2020) [172]	Italy	1252	64.7±15.5	798 (63.74)	Retrospective, observational, single-center study	CAD	124 (9.9)	HR 1.14 (0.79-1.63)	Death	Age, gender, LVEF<35%, CVA, atrial fibrillation, diabetes mellitus, hypertension, smoking, CKD	5
Grodecki et al. (2021) [175]	USA	109	63.74±15.11	68 (62.39)	Prospective	Heart failure	16 (14.68)	OR 3.5 (1.1-8.2)	Death	Age, gender, diabetes mellitus, hypertension, smoking history, chronic lung disease, history of coronary artery disease, epicardial adipose tissue volume (mL), epicardial adipose tissue attenuation, total pneumonia burden	7
Rossi et al. (2020) [80]	Italy	590	76.2 (68.2–82.6)	399 (67.6)	Retrospective observational study	CVD	95 (16.1)	HR 1.180 (0.855–1.628)	Mortality	Age, gender, vital signs at admission (temperature, PaO2/FiO2, PaO2/FiO2<300), laboratory parameters (LDH, CRP, white blood cell count, lymphocyte’s rate), chronic diseases (hyperlipidemia, diabetes, atrial fibrillation, COPD, CKD, stroke, malignancy, 3 or more comorbidities), chronical drugs intake (ACEi, ARBs, CCBs, Alpha blockers, Diuretics, Beta blockers)	6
Khan et al. (2020) [177]	Saudi Arabia	648	34±19	342 (52.8)	Retrospective cohort study	Cardiac diseases	23 (3.5)	OR 3.05 (1.16-8.02)	ICU admission	Age, gender, smoker, comorbidities (one or more comorbidity, two or more comorbidity, diabetes mellitus, HTN, CRD, chronic kidney diseases, cancer/immunodeficiency), symptoms (fever, cough, sore throat, runny nose, headache, GI symptoms, myalgia), vital signs (temperature (≥38), heart rate ≥100, respiratory rate, respiratory rate, respiratory rate, DBP, oxygen saturation, oxygen saturation)	7
Rutten et al. (2020) [178]	Netherlands	1538	84±8.7	554 (36.02)	Prospective cohort study	CVD	53 (3.47)	HR 1.15 (0.97-1.35)	Mortality	Age, gender, comorbidity (Dementia, cerebrovascular disease, diabetes mellitus, chronic respiratory disease, reduced kidney function, Parkinson’s disease)	6
Schuelter-Trevisol et al. (2020) [179]	Brazil	211	51.2*	113 (53.6)	Cohort study	Chronic heart disease	27 (12.9)	OR 0.98 (0.31-3.10)	Death	Age, gender, comorbidities (arterial hypertension, diabetes mellitus, obesity, neurologic/psychiatric diseases, chronic lung diseases, dyslipidemia, smoking habits, cancer, chronic kidney diseases, vascular diseases)	6
FAI2R /SFR/SNFMI/SOFREMIP/CRI/IMIDIATE (2020) [174]	France	694	56.1±16.4	232 (33.4)	Observational, multicenter, French national cohort study	Coronary heart diseases	68 (9.8)	OR 1.86 (0.97–3.56)	Severity	Age, gender	8
Nyaberaet al. (2020) [181]	USA	290	77.6±8.3	150 (51.7)	Single-center retrospective cohort study	CAD	80 (27.6)	OR 0.91 (0.52-1.62)	Mortality	BMI, age, COPD, asthma, DM, HTN, end-stage renal disease	4
Ozturk et al. (2021) [182]	Turkey	1160	60.5 (47–71)	627 (54.1)	Multicenter, retrospective, observational study	CVD	NR (NR)	HR 1.242 (0.850–1.815)	Death	Age, gender, diabetes mellitus, HTN, COPD, albumin, hemoglobin, lymphocyte count, platelet count, CRP increase, clinic presentation, COVID-19 diagnosis by RT-PCR, patient group, control group (HD group, RT group, CKD group)	5
Druyan et al. (2021) [183]	Israel	181	62.71*	107(59.1)	Single center study	Heart failure	10 (5.52)	OR 2.35 (0.24-18.64)	Severe, critical or fatal COVID19	Gender, AID, HTN, dyslipidemia, diabetes, malignancy, IHD, arrhythmia, obesity, pulmonary disease, smoking, CVA, renal failure, older age	5
Alguwaihes et al. (2020) [184]	Saudi Arabia	439	55 (19–101)	300 (68.3)	Single-center retrospective study	CVD	44 (10.0)	HR 1.8 (0.7–4.4)	Death	Age, gender, comorbidities (obesity, HTN, diabetes mellitus, chronic kidney disease, congestive heart failure, stroke, smoking), medications (β-Blocker use, ACE inhibitor use, ARB Use), laboratory investigations (RBG, FPG, HbA1c>9.0%, bilateral lung infiltrates, neutrophil count>7.5, creatinine>90 μmol/l, ALT>65 U/l, 25(OH)D<12.5 nmol/l)	7
Özdemir et al. (2021) [185]	Turkey	101	49.60±18	55 (54.4)	Retrospective study	Chronic heart failure	10 (9.9)	HR 1.02 (0.98 – 1.10)	QTc prolongation	Baseline QTc, HCQ alone, HCQ + AZM	6
Gue et al. (2020) [186]	UK	316	73.42±15.97	192 (61.1)	Single-center retrospective cohort	CAD	48 (15.19)	OR 1.62 (0.76–4.07)	30-day mortality	Age, gender, HTN, atrial fibrillation, oral anticoagulants, modified sepsis-induced coagulopathy score	7
Galiero et al. (2020) [187]	Italy	618	65±15.2	379 (61.3)	Multicenter retrospective observational cohort study	Chronic Cardiac Disease	166 (26.9)	OR 0.96 (0.53-1.76)	Mortality	Age, gender, Glasgow Coma Score/15, respiratory severity Scale, CKD, CLD, chronic respiratory disease, malignancies	6
Rosenthal et al. (2020) [188]	USA	64781	56.1±19.9	31968 (49.3)	Retrospective cohort study	Myocardial infarction	3717 (5.7)	OR 1.47 (1.34-1.62)	In-Hospital Mortality	Age, gender, race, payer type, admission point of origin, hospital region, hospital beds, hospital teaching status, hospital teaching status, Sepsis, acute kidney failure, hypokalemia, acidosis, acute liver damage, neurological disorder, baseline comorbidities (Cerebrovascular disease, COPD, dementia, diabetes, any malignant neoplasm, metastatic solid tumor, hemiplegia, AIDS, HTN, Hyperlipidemia)	9
Rethemiotaki et al. (2020) [189]	the World Health Organization dataset and Chi nese Center for Disease Control and Preventio	44672	71*	22981 (51.44)	NR	CVD	92 (15.9)	OR 13.6 (10.3–17.9)	Death	Age, gender, occupation (service industry, farmer/laborer, health worker, retiree, other/none), province: (Hubei, Other), Wuhan-related exposure, comorbid condition (HTN, diabetes, chronic respiratory disease, cancer (any), none)	8
Pantea Stoian et al. (2020) [176]	China	432	NR	NR	Multiple-case, multiple-center	Heart failure	30 (6.94)	OR 2.990 (1.612–5.546)	Death	Age, gender, HTN, obesity, diabetes type 2, dialysis, chronic kidney disease, COPD, supraventricular tachyarrhythmia, respiratory failure, Intercept	7
Zhou et al. (2020) [191]	China	134	62.08±14.38*	85 (63.4)	Retrospective	Coronary heart disease	16 (11.94)	OR 1.098 (0.202–5.959)	Death	Gender, age, HTN, coronary heart disease, neutrophil, lymphocyte, ALT, IL-2, IL-6, TNF-α, D-dimer, and total CT score	6
Stefan et al. (2021) [192]	Romania	37	64 (55–71)	19 (51)	Retrospective, observational, single-center study	Coronary heart disease	19 (51.0)	HR 0.98 (0.05–17.54)	In-hospital death	Age, hemodialysis vintage, obesity, current smoker, diabetes mellitus, Charlson comorbidity index, basal oxygen saturation, hemoglobin, lymphocytes, CRP, serum albumin, LDH, Lopinavir–ritonavir, Tocilizumab, hydroxychloroquine, glucocorticoids	7
Ahnach et al. (2021) [180]	Morocco	101	50 (32–63)	75 (51.72)	Retrospective study	CVD	16 (11.03)	OR 3.74 (0.76–18.29	Disease severity	Age, gender, HTN, diabetes, other disease, respiratory symptom, neutrophil, lymphocyte, eosinophil, CRP	6
Eshrati et al. (2020) [193]	Iran	3188	55.05 ± 0.31	1925 (60.4)	Retrospective cohort study	CVD	401 (12.6)	HR 0.60 (0.83-1.13)	death	Age, gender, immune disease, diabetes, liver disease, kidney disease, ,COPD, cancer, chronic nervous disease, type of treatment	8
Özyılmaz et al. (2020) [194]	Turkey	105	45 (20–87)	76 (72.3)	Single-center, retrospective, observational study	CAD	14 (13.3)	OR 0.024 (0.000–1.207)	Mortality	Troponin I, C-Reactive protein, lymphocyte count, shortness of breath, HTN, hyperlipidemia, diabetes mellitus	7
Tan et al. (2020) [195]	China	163	69.0 (62.0-78.0)	109 (66.9)	Retrospective study	Chronic cardiac injury	25 (15.3)	OR 2.660 (1.034-6.843)	Mortality	Age, gender, HTN, diabetes	5
Ling et al. (2020) [196]	UK	444	74 (63-83)	245 (55.2)	Cross-Sectional Multi-Centre Observational Study	Heart failure	54 (12.2)	OR 1.61 (0.87–2.99)	Mortality	Age, gender, diabetes, non-Caucasian ethnicity, baseline serum 25(OH)D levels, vitamin D deficiency, treatment with cholecalciferol booster therapy, admission SpO2 < 96%, admission CRP > 73 mg/L, admission creatinine > 83 μmol/L, received CPAP, length of stay >11 days, diabetes (types 1 and 2 combined), admission glucose > 6·9 mmol/L, COPD, asthma, IHD, current or previous ACS, HTN, current or previous TIA or stroke, dementia, obesity, malignancy of solid organ, malignancy of skin, hematological malignancy, solid organ transplant, inflammatory arthritis, inflammatory bowel disease	5
Zhong et al. (2020) [197]	China	126	66.3±10.6	56 (44.4)	Retrospective observational study	CVA	21 (16.7)	OR 2.03 (0.45-9.08)	Death	Age, gender, ACEI/ARB, stains	5
Izurieta et al. (2020) [198]	USA	12613	80.5*	6496 (51.5)	Retrospective cohort study	Congestive Heart Failure	3557 (28.2)	OR 1.30 (1.23, 1.36))	Death	Age, gender, reason for entering medicare, ADI national rank, logged COVID-19 circulation rate by 100,000, logged population density by county, vaccination, presence of medical conditions (HTN, obesity, diabetes, hospitalized stroke/TIA, coronary revascularization, atrial fibrillation, hospitalized AMI, other cerebrovascular disease, COPD, asthma without COPD, interstitial lung disease, hypersensitivity pneumonitis, bronchiectasis, chronic liver disease, neurological/neurodevelopmental conditions), frailty conditions, immunocompromised status, estimated overall, interaction effects of age, dual-eligibility, and race, 80 years old vs. 65 years old, dual-eligible vs. non-dual-eligible, dual-eligible vs. non-dual-eligible, effects of being dual-eligible, by race, non-whites vs. whites, non-dual-eligible, non-whites vs. whites, dual-eligible	8
Burrell et al. (2021) [199]	Australia	304	63.5 (53–72)	140 (69%)	Prospective, observational cohort study	Chronic cardiac disease	40 (20)	HR 3.38 (1.46–7.83)	Mortality	Age, gender, APACHE-II score on ICU day 1, comorbid conditions (comorbid conditions),	5
Li et al. (2020) [190]	China	123	64.43±14.02	62 (50.41)	Retrospective study	CVD	26 (21.14)	OR 0.686 (0.227–2.076)	Unfavorable clinical outcomes	Age, gender, diabetes, HTN, COPD, CT severity score, GGO volume, GGO volume percentage, consolidation volume, consolidation volume percentage	4
Caliskan et al. (2020) [200]	Turkey	56	48±19.664	NR	Retrospective observational study	CAD	42 (7.4)	OR 6.252 (2.171-18.004)	Mortality	Former smoker, current smoker, age, COPD, diabetes, dementia, HTN, chronic renal failure, arrhythmia	5
Vafadar et al. (2021) [201]	Iran	219	57.8±16.5	137 (62.6)	Retrospective cohort	Ischemic heart disease	46 (22.37)	HR 1.98 (0.94–4.17)	Mortality	Respiratory rate, SpO2 ≤ 90%, WBC count, NLR, age	6
Working group for the surveillance and control of COVID-19 in Spain et al. (2020) [202]	Spain	2612	83 (75–89)	14680 (56.2)	NR	CVD	11444 (59.9)	OR 1.32 (1.23-1.42)	Death	Gender, age, pneumonia, acute respiratory distress syndrome, acute renal failure, Diabetes, HTN, chronic lung disease, chronic renal disease, healthcare worker	6
Rashidi et al. (2021) [203]	Iran, Germany, USA	1529	56 (32–80)	832 (54.4)	Multi-center prospective study	Cardiac disease	149 (9.7)	OR 0.80 (0.36–1.76)	Death	Age, gender, recent cancer, COPD, CKD, smoking, diabetes mellitus, HTN	5
Chaudhri et al. (2020) [204]	USA	317	59.16±17.5	166 (52.37)	Single-center cohort study	Coronary artery disease	27 (12)	OR 0.92 (0.39-2.17)	Key outcomes	Age, gender, history of ARB use,history of ACEI use, HTN, diabetes, CKD	5
Huh et al. (2021) [205]	South Korea	219961	49.4 (18–116)	104331 (47.4)	Retrospective case-control study	Chronic heart disease	32457 (14.76)	OR 1.31 (1.04-1.65)	The requirement of any one of the following or death: supplementary oxygen, high-flow nasal cannula, non-invasive ventilation, mechanical ventilation, and extracorporeal membrane oxygenation	Drugs commonly used for chronic conditions (angiotensin receptor blockers, angiotensin converting enzyme inhibitors, metformin, thiazolidinedione, Statins, NSAIDs), drugs with potential therapeutic effect, drugs with potential therapeutic effect, comorbidities (Charlson comorbidity index, mean (SD), Diabetes, HTN, chronic lung disease, asthma and allergic rhinitis, chronic liver disease, Chronic kidney disease, Malignancy, RA, SLE, GCA, and JIA, other connective tissue disease, chronic neurologic disease, Pancreatitis), healthcare utilization	8
Orioli et al. (2021) [206]	Belgium	73	69±14	48 (66.67)	Retrospective study	CVD	32 (43.8)	HR 3.54 (1.60-7.82)	In-hospital death	Diabetes, cognitive impairment, area of lung injury >50%	6
Gude-Sampedro et al. (2021) [207]	Spain	10454	58.0±20.0	4172 (39.9)	Retrospective cohort study	Ischemic heart disease		OR 1.61 (1.20-2.33)	Death	Age, gender, lymphoma/leukemia, dementia, COPD, diabetes, chronic kidney disease	9
Monteiro et al. (2020) [208]	USA	112	61 (45–74)	74 (66)	Retrospective, observational cohort study	CAD	17 (15)	OR 0.48 (0.08–3.08)	Requiring mechanical ventilation	Age, gender, past medical history (obesity, diabetes, HTN, CKD), Tobacco exposure history	4
Lano et al. (2020) [209]	France	122	73.5 (64.2–81.2)	79 (65)	Observational cohort multicenter study	Congestive heart failure	13 (11)	OR 1.222 (0.309–4.649)	Mortality	Age, atrial fibrillation, ARBs (current medication)	8
Lanini et al. (2020) [210]	Italy	379	61.67±15.60	273 (72.03)	Longitudinal cohort study	CVD	19 (5.01)	OR 2.79 (1.29-6.03)	Death	Age, gender, diabetes, neoplasm, obesity, chronic renal failure, COPD	4
Schwartz et al. (2020) [212]	Canada	56606	31*	29205 (51.59)	Cross-sectional study	CVD	4465 (7.89)	OR 1.10 (0.99–1.22)	Death	Healthcare worker, age, comorbidities (asthma, COPD, renal conditions, diabetes, immune compromise or cancer, obesity, other medical conditions None), exposed to long-term care home, symptoms (fever and/or cough, other symptoms, missing, asymptomatic)	9
Sun et al. (2021) [213]	China	3400	61 (50-68)	1649 (48.5)	Retrospective cohort study	CVD	343 (10.1)	OR 2.85 (1.65-4.94)	Death	Comorbid conditions (Neither HTN nor T2DM, Hypertension alone, T2DM alone, HTN and T2DM), age, gender, cerebrovascular disease, chronic kidney disease, chronic liver disease, chronic lung disease, endocrine/Immune system disease, tumor, ACEIs/ARBs treatment	6
McGurnaghan et al. (2021) [214]	Scotland	319349	79.9 (71.4–85.7)	180486 (56.5)	Cohort study	Any heart disease	696 (64.3)	OR 2.425 (2.135–2.754)	Fatal or critical care unit-treated COVID-19	Sociodemographic (age, gender, diabetes type, diabetes duration, care home resident, any hypoglycemia admission in past 5 years, deprivation index, ethnicity, comorbidities (any diabetic ketoacidosis admission in past 5 years, any hypoglycemia admission in past 5 years, ever admitted to hospital in past 5 years, asthma or chronic lower airway disease, neurological and dementia (excluding epilepsy),liver disease, immune disease or on immunosuppressants, any listed condition), other clinical measures (insulin pump use, flash glucose monitor use, HbA1c, BMI, systolic blood pressure, diastolic blood pressure, total cholesterol, Estimated glomerular filtration rate, albuminuria grade, retinopathy grading, tobacco smoking), drug exposures (any lipid lowering, any proton pump inhibitor, any non-steroidal anti-inflammatory drugs, any anticoagulants, Any antihypertensive, number of ATC level 3 drug classes (excluding for diabetes), number of diabetes drug classes prescribed)	8
Cetinkal et al. (2020) [215]	Turkey	349	68.3±13.3	176 (50.43)	Retrospective single-center study	Heart failure	38 (10.89)	OR 2.40 (0.82-7.01)	In-hospital mortality	Neutrophil to lymphocyte ratio, gender, age, diabetes mellitus, Use of RAAS blockers, chronic kidney disease, Smoking, COPD, d-dimer, LDH, procalcitonin, Ferritin	6
Xu et al. (2020) [216]	China	61	63.62±10.78	33 (54.1)	Retrospective	Heart diseases	7 (11.5)	OR 2.94 (0.42-21.78)	Severity	Age, gender, diabetes, HTN, hepatic dysfunction, mild-nonlung involvement	4
Lv et al. (2021) [217]	China	409	50.47±12.43	188 (46)	Retrospective cohort Study	Heart disease	51 (12.5)	HR 2.650 (1.079–6.510)	Death	Age, gender, fever, cough, sputum, tiredness, body aches, diarrhea, number of symptoms, HTN, diabetes, pulmonary disease, other comorbidities, CT ground-glass, opacity, CT bilateral pulmonary infiltration	5
Guerra et al. (2021) [218]	Spain	447	55.0±22.5	190 (46.4)	Retrospective single center study	Coronary artery disease		OR 4.95 (1.51-16.27)	Mortality	Gender, HTN, COPD, cancer, diabetes, obesity, CLD, age	6

*, studies included 2 two different cohort samples; *HTN* Hypertension, *SOFA* sequential organ failure assessment, *ALT* alanine aminotransferase, *AST* aspartate aminotransferase, *ARDS* acute respiratory distress syndrome, *INR* international normalized ratio, *ICU* intensive care unit, *HF* heart failure, *IL-8* interleukin-8, *AKI* acute cardiac injury, *CLD* chronic lung diseases, *CRD* chronic renal disease, *CKD* chronic kidney disease, *IL-6* interleukin-6, *WBC* white blood cell, *NR* not reported, *HTN* hypertension, *HR* hazard ratio, *OR* odds ratio, *CI* confidence interval, *CHD*, coronary heart disease, *CVD* cardiovascular disease, *CAD* coronary artery disease, *CKD* chronic kidney diseases, *CLD* chronic liver diseases, *COPD* chronic obstructive pulmonary disease, *CRP* C-reactive protein, *hs-CRP* high-sensitivity C-reactive protein, *BMI* body mass index, *LYM%* lymphocyte percentage, *NEU%* neutrophil percentage, *NLR* ratio of neutrophil to lymphocyte, *FIB* fibrinogen content, *TBIL* total bilirubin, *ALB* albumin, *Cr* creatinine, *GFR* glomerular filtration rate, *CK-MB* creatine kinase isoenzyme-MB, *CT* computerized tomography, *PCT* procalcitonin, *GGO* ground-glass opacity, *ICI* immune check point inhibitors, *HCQ* hydroxychloroquine, *AZM* azithromycin, *APTT* activated partial thromboplastin time, *ACE* angiotensin converting enzyme inhibitors, *ARB* angiotensin II receptor blockers, *eGFR* estimated glomerular filtration rate, *PAD* peripheral arterial disease, *Hb* hemoglobin, *LDH* lactate dehydrogenase, *ESR* erythrocyte sedimentation rate, *MYO* myoglobin, *LFTs* liver function tests, *SABA* short acting beta agonists, *ESRD* end-stage renal disease (on dialysis), *ALC* absolute lymphocyte count, *ANC* absolute neutrophil count, *MV* mechanical ventilation, *APACHE II* acute physiology and chronic health evaluation II, *BUN* blood urea nitrogen, *CVA* cerebrovascular accident, *TIA* transient ischemic attack, *DBIL* direct bilirubin, *IBIL* indirect bilirubin, *PT* prothrombin time, *FBG* fasting blood glucose.

**Table 2:** The results of subgroup analysis

Variables	Effects	NO. Of studies	Subgroup analysis		Prediction interval
			Pooled ES (95% CI)	*I²,Tau², P* value	
Sample size					
>=1000	HR	24	1.16 (1.03-1.32)	*I² = 88%, τ² = 0.0697,P < 0.01*	*0.66-2.04*
	OR	53	1.41 (1.32-1.51)	*I² = 84%, τ² = 0.0694,P < 0.01*	*0.84-2.39*
<1000	HR	41	1.63 (1.41-1.88)	*I² = 64%, τ² = 0.0957,P < 0.01*	*0.86-3.10*
	OR	83	1.57 (1.40-1.77)	*I² = 57%, τ² = 0.0967, P < 0.01*	*0.84-2.95*
Age					
>=60	HR	41	1.42 (1.25-1.61)	*I² = 73%,τ² = 0.0914, P < 0.01*	*0.76-2.65*
	OR	78	1.49 (1.34-1.65)	*I² = 86%, τ² = 0.1144,P < 0.01*	*0.75-2.95*
<60	HR	23	1.18 (1.04-1.33)	*I² = 81%, τ² = 0.0181,P < 0.01*	*0.77-1.80*
	OR	58	1.30 (1.19-1.42)	*I² = 76%, τ² = 0.0379,P < 0.01*	*0.87-1.94*
NR	HR	1	2.59 (1.16-5.79)	-	-
	OR	2	1.75 (0.67-4.61)	*I² = 88%, τ² = 0.4301,P < 0.01*	*-*
Male (%)					
>=50	HR	44	1.41 (1.23-1.60)	*I² = 83%, τ² = 0.1123,P < 0.01*	*0.71-2.80*
	OR	94	1.33 (1.23-1.44)	*I² = 78%, τ² = 0.0558,P < 0.01*	*0.83-2.14*
<50	HR	21	1.25 (1.13-1.38)	*I² = 55%, τ² = 0.0179,P < 0.01*	*0.92-1.69*
	OR	36	1.42 (1.27-1.58)	*I² = 56%, τ² = 0.0431,P < 0.01*	*0.92-2.20*
NA	HR	0	-	-	-
	OR	8	2.25 (0.87-5.79)	*I² = 98%, τ² = 1.6735,P < 0.01*	*0.08-65.97*
Study design					
Retrospective/case series	HR	38	1.50 (1.30-1.73)	*I² = 81%, τ² = 0.1067,P < 0.01*	*0.76-2.96*
	OR	88	1.37 (1.28-1.47)	*I² = 65%, τ² = 0.0269,P < 0.01*	*0.98-1.91*
Prospective study	HR	9	1.11 (0.74-1.67)	*I² = 88%, τ² = 0.2724,P < 0.01*	*0.28-4.39*
	OR	7	1.31 (0.84-2.06)	*I² = 77%, τ² = 0.2451,P < 0.01*	*0.32-5.34*
Others	HR	19	1.25 (1.12-1.39)	*I² = 63%, τ² = 0.0214,P < 0.01*	*0.90-1.74*
	OR	43	1.45 (1.24-1.70)	*I² = 93%, τ² = 0.1725,P < 0.01*	*0.62-3.42*
Region					
Europe	HR	27	1.31 (1.17-1.47)	*I² = 83%,τ² = 0.0462, P < 0.01*	*0.83-2.08*
	OR	54	1.47 (1.33-1.64)	*I² = 75%, τ² = 0.0725,P < 0.01*	*0.85-2.56*
North America	HR	12	1.16 (1.02-1.33)	*I² = 52%,τ² = 0.0234, P = 0.02*	*0.80-1.69*
	OR	42	1.18 (1.08-1.29)	*I² = 77%, τ² = 0.0333,P < 0.01*	*0.81-1.72*
Asia	HR	24	1.64 (1.24-2.16)	*I² = 81%,τ² = 0.3015, P < 0.01*	*0.51-5.30*
	OR	37	1.55 (1.29-1.87)	*I² = 68%, τ² = 0.1272,P < 0.01*	*0.73-3.29*
Others	HR	2	2.12 (0.89-5.01)	*I² = 59%, τ² = 0.2289,P = 0.12*	*-*
	OR	5	3.54(0.86-14.60)	*I² = 92%, τ² = 2.2249,P < 0.01*	*0.02-691.66*
Disease					
CVD	HR	27	1.36 (1.15-1.61)	*I² = 79%, τ² = 0.1154,P < 0.01*	*0.66-2.80*
	OR	41	1.48 (1.24-1.76)	*I² = 91%, τ² = 0.1984,P < 0.01*	*0.59-3.70*
Cardiac disease	HR	25	1.40 (1.17-1.69)	*I² = 77%, τ² = 0.1141,P < 0.01*	*0.68-2.90*
	OR	38	1.43 (1.25-1.64)	*I² = 84%, τ² = 0.0762,P < 0.01*	*0.80-2.55*
HF	HR	4	1.23 (1.05-1.44)	*I² = 89%, τ² = 0.0173,P < 0.01*	*0.63-2.39*
	OR	31	1.46 (1.31-1.62)	*I² = 59%, τ² = 0.0290,P < 0.01*	*1.01-2.10*
CAD	HR	9	1.48 (1.14-1.93)	*I² = 70%, τ² = 0.0957,P < 0.01*	*0.67-3.29*
	OR	26	1.17 (1.02-1.35)	*I² = 52%,τ² = 0.0416, P < 0.01*	*0.75-1.83*
Others	HR	-	-	*-*	
	OR	2	1.63 (1.05-2.53)	*I² = 33%, τ² = 0.0585,P = 0.22*	*-*
Outcomes					
Mortality	HR	55	1.39 (1.27-1.53)	*I²* = 76%, *τ² = 0.0597,*P < 0.01	*0.85-2.30*
	OR	98	1.44 (1.32-1.56)	*I² = 84%, τ² = 0.0840, P < 0.01*	*0.80-2.57*
Severity	HR	7	1.06 (0.70-1.60)	*I² = 88%, τ² = 0.2418,P < 0.01*	*0.30-3.68*
	OR	25	1.22 (1.03-1.43)	*I² = 66%, τ² = 0.0575,P < 0.01*	*0.72-2.06*
Disease progression	HR	3	1.65 (1.20-2.27)	*I² = 0%, τ² = 0.000,P = 0.56*	*0.21-12.92*
	OR	15	1.63 (1.31-2.04)	*I² = 68%, τ² = 0.0858,P < 0.01*	*0.84-2.39*

Note: ES, effect sizes; CI, confidence interval; OR, odds ratio; HR, hazards ratio.

**Fig. 2:**
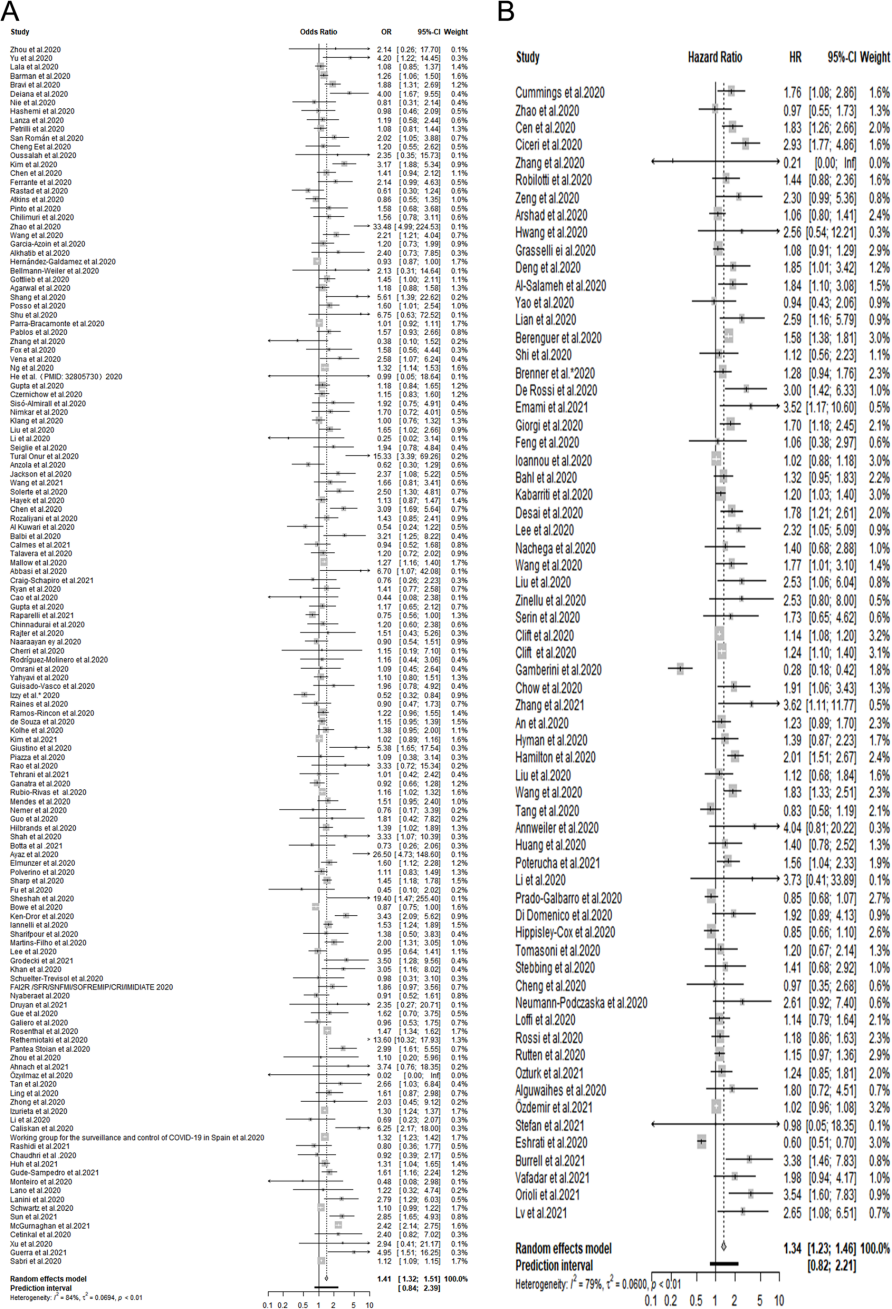
Forest plot of adjusted pooled effects for adverse outcomes associated with CVD in patients with COVID-19. **A**) Pooled OR; **B**) Pooled HR

**Fig. 3:**
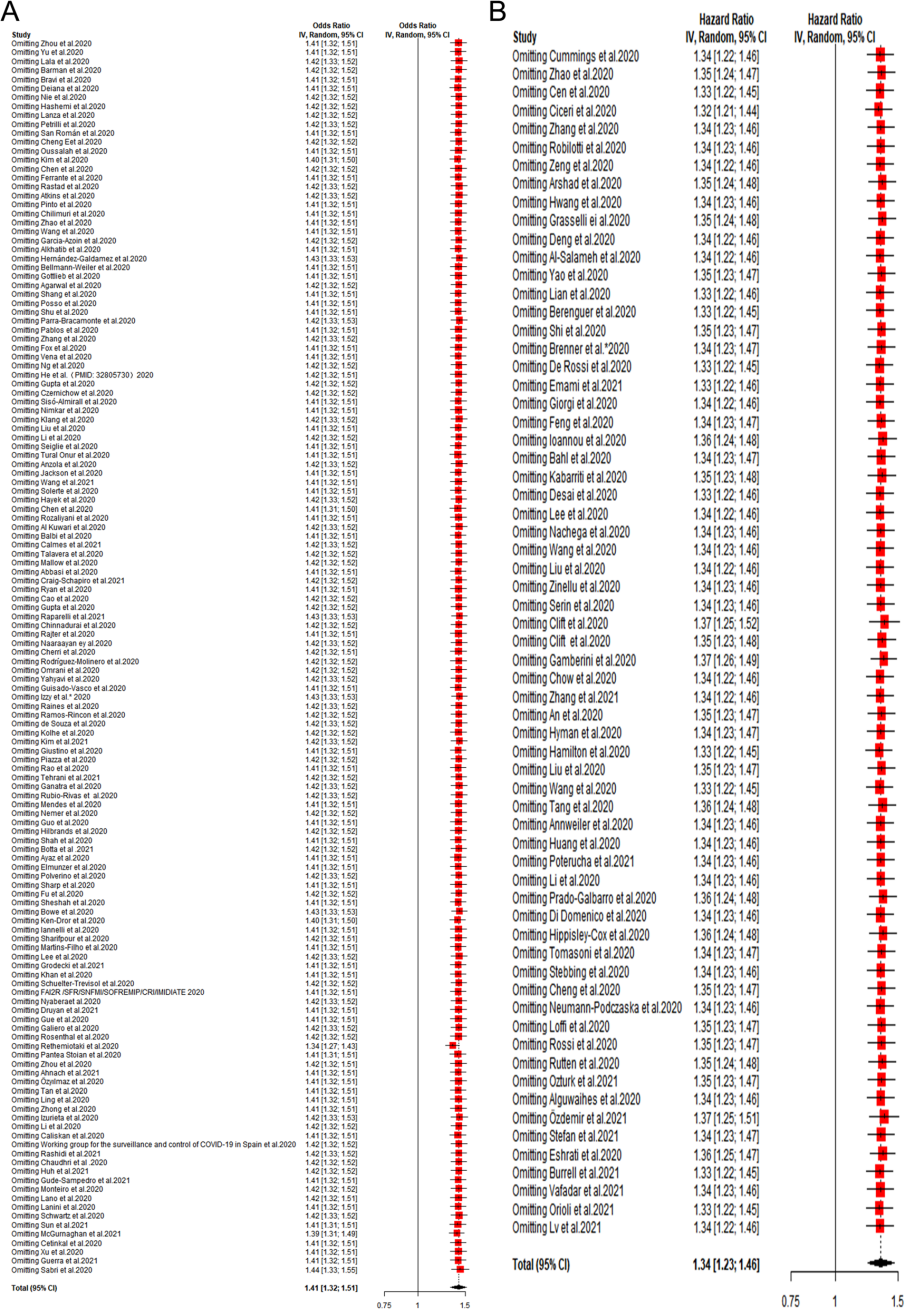
Sensitivity analysis for pooled OR (**A**) and HR (**B**)

**Fig. 4:**
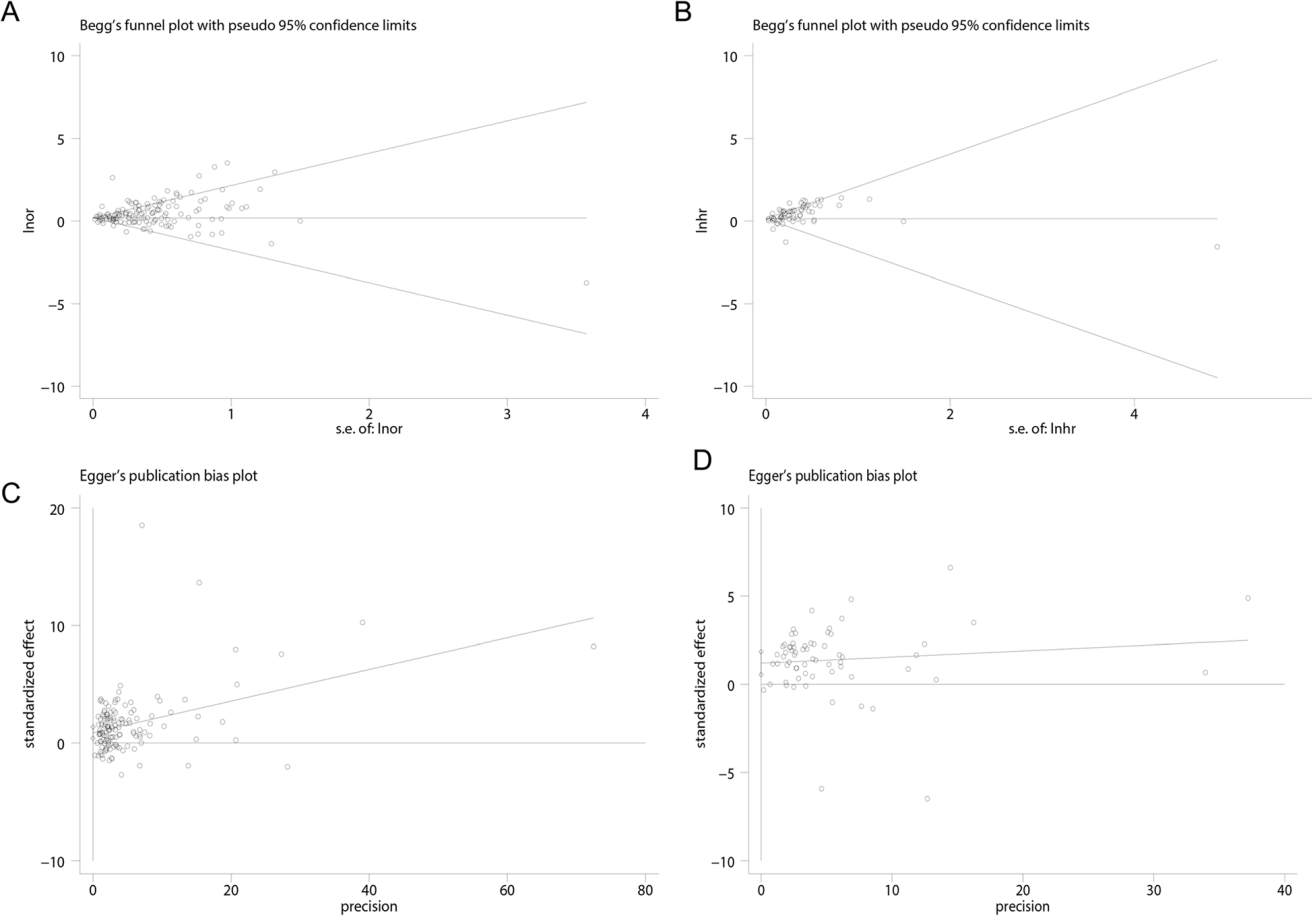
Publication bias for pooled OR (**A** and **B**) and HR (**C** and **D**)

Totally, our results revealed that COVID-19 patients who suffered from CVD tended more to adverse outcomes (pooled ORs = 1.41, 95% CIs: 1.32-1.51, prediction interval: 0.84-2.39; pooled HRs = 1.34, 95% CIs: 1.23-1.46, prediction interval: 0.82-2.21 Fig. 2). Subgroup analysis by sample size showed consistent results (pooled HRs = 1.16, 95% CIs: 1.03-1.32, prediction interval: 0.66-2.04; pooled ORs = 1.41, 95% CIs: 1.32-1.51, prediction interval: 0.84-2.39 for sample size >= 1000; pooled HRs = 1.63, 95% CIs: 1.41-1.88, prediction interval: 0.86-3.10; pooled ORs: 1.57, 95% CIs: 1.40-1.77, prediction interval: 0.84-2.95 for sample size < 1000; Table 2 and Fig. A1). The positive association between pre-existing CVD and adverse outcomes in COVID-19 patients was also observed in subgroup analysis by disease types (Table 2 and Fig. A2): cardiac disease (pooled HRs = 1.40, 95% CIs: 1.17-1.69, prediction interval: 0.68-2.90; pooled ORs = 1.43, 95% CIs: 1.25-1.64, prediction interval: 0.80-2.55), HF (pooled HRs = 1.23, 95% CIs: 1.05-1.44, prediction interval: 0.63-2.39; pooled ORs = 1.46, 95% CIs: 1.31-1.62, prediction interval: 1.01-2.10), and CAD (pooled HRs = 1.48, 95% CIs: 1.14-1.93, prediction interval: 0.67-3.29; pooled ORs = 1.17, 95% CIs:1.02-1.35, prediction interval: 0.75-1.83). In addition, subgroup analyses stratified by age, the proportion of males, region, disease outcomes and study design supported the above positive associations (Table 2 and Fig. A3-7). Sensitivity analysis indicated that our result was robust (Fig. 3A and B). There was no publication bias was detected by Begg’s test (OR: *P* = 0.233, HR: *P* = 0.054; Fig. 4A and B), while significant publication bias was found by Egger’s test (OR: *P* = 0.000, HR: *P* = 0.000; Fig. 4C and D). Therefore, the trim-and-fill method was adopted for further analysis. The results for HR showed that with the addition of 21 more studies, the results of the meta-analysis would be more robust but not reversed (pooled HRs = 1.11, 95% CIs: 1.01-1.14, fixed-effects model; pooled HRs = 1.16, 95% CIs: 1.06-1.26, random-effects model), and the OR results (pooled ORs: 1.18, 95% CIs: 1.16-1.20, fixed-effects model; pooled ORs: 1.21, 95% CIs: 1.12-1.30, random-effects model) showed that the results would be equally robust after adding 29 studies. However, there was high heterogeneity in our study. To find sources of heterogeneity, we conducted a meta-regression. However, adjustments for multivariate regression coefficients for sample size, age, proportion of males, study design, region, disease types, disease outcomes were not statistically significant (Table 3), suggesting that these were not sources of heterogeneity identified.

**Table 3:** The result of meta-regression

Variables	HR			OR		
	Tau²	t-value	P-value	Tau²	t-value	P-value
Sample size	0.0753	-0.3248	0.0007	0.0931	-0.1552	0.0449
>=1000						
<1000						
Age	0.0552	-	0.1123	0.0746	-	0.3495
>=60		0.1404	0.1206		0.1006	0.1674
<60						
NR		0.7562	0.1143		0.1713	0.5027
Male (%)	0.0734	0.0351	0.7253	0.0997	-	0.0086
>=50					-0.0678	0.4355
<50						
NR					0.4272	0.0119
Study design	0.0774	-	0.0828	0.0796	-	0.8863
Retrospective/case series		0.1064	0.3152		-0.0034	0.9647
Prospective study		0.1064	0.1628		-0.0823	0.6301
Others						
Region	0.0651	-	0.1800	0.0601	-	<0.0001
Europe		-0.1169	0.2910		-0.0307	0.7439
North America		-0.2287	0.0746		-0.2362	0.0132
Asia						
Others		0.3260	0.3447		1.3471	<0.0001
Disease	0.0702	-	0.8655	0.1005	-	0.4005
CVD		-0.1123	0.4286		0.1737	0.1365
Cardiac disease		-0.0681	0.6418		0.1620	0.1741
HF		-0.1221	0.5212		0.2230	0.0640
CAD						
Others					0.82	0.413
Outcomes	0.0694	-	0.0375	0.0810	-	0.1400
Mortality		-0.0990	0.6880		-0.1298	0.2733
Severity		-0.4713	0.0915		-0.2786	0.0528
Disease progression

## Discussion

Many countries have been hit by the pandemic caused by SARS-CoV-2, numerous people lost their lives because of this. Meanwhile, health systems in every country were under so unprecedented strain that it was very important to find an effective marker to help implement bed grading management. What called for special attention was that earlier studies have shown COVID-19 patients with at least one underlying conditions, such as chronic kidney disease, HIV, diabetes and other comorbidities, have a poor disease course [2, 29, 211, 219, 220], which means that those patients with underlying diseases should be monitored more carefully in case of disease getting worse. Furthermore, it was reported that the risk of primary respiratory syndrome severity and adverse outcomes was increased in Middle East respiratory syndrome (MERS) patients with pre-existing CVD. The research by Li et al. [8] with unadjusted effect estimates showed that there was a positive association between CVD and adverse outcomes in patients with COVID-19, but the association might be confounded by other factors such as age, gender and comorbidities. Thus, we performed a quantitative meta-analysis on the basis of adjusted effect estimates to clarify whether pre-existing CVD was an independent risk factor associated with adverse outcomes in COVID-19 patients.

Our results based on adjusted effect estimates revealed that pre-existing CVD was significantly related to adverse outcomes in COVID-19 patients on the basis of 203 eligible studies with 24,032,712 cases. The significant association between pre-existing CVD and adverse outcomes in COVID-19 patients was still existent in further subgroup analyses stratified by the proportion of males, study design, disease types, sample size, region and disease outcomes, which suggests that our findings are relatively stable.

Similar to other meta-analyses, several limitations should be acknowledged in this present study. Firstly, data on drug and supportive treatments are not clear in the selected studies presently, thus, we could not evaluate the effects of treatments on the association between co-existing CVD and adverse outcomes in COVID-19 patients. Secondly, statistically significant results were more likely to be accepted and published than non-statistically significant results in similar studies, but in fact, the data of the meta-analysis mainly derived from the studies which have been published, which may lead to publication bias. Thirdly, the causal relationship of CVD and adverse outcomes in patients with COVID-19 cannot be confirmed on account of the inherent limitation of the observational study. Therefore, well-designed studies with larger sample sizes are needed for further verification.

## Conclusions

In conclusion, our findings indicated that pre-existing CVD was an independent risk factor associated with adverse outcomes among COVID-19 patients. COVID-19 patients with a history of CVD might need more attention.

## Supplementary Information

**Additional file 1: Table A1.** Preferred Reporting Items for Systematic Reviews and Meta-analysis (PRISMA) guidelines. **Fig. A1.** Subgroup analysis stratified by sample size. **Fig. A2.** Subgroup analysis stratified by type of disease. **Fig. A3.** Subgroup analysis stratified by age. **Fig. A4.** Subgroup analysis stratified by the proportion of male. **Fig. A5.** Subgroup analysis stratified by study design. **Fig. A6.** Subgroup analysis stratified by region. **Fig. A7.** Subgroup analysis stratified by outcome of disease.

## Data Availability

All data relevant to the study are included in the article or uploaded as supplementary information.

## Abbreviations

CVD: Cardiovascular disease
COVID-19: Coronavirus disease 2019
CI: Confidence interval
OR: Odds ratio
HR: Hazard ratio
CHD: Coronary heart disease
CAD: Coronary artery disease
HIV: Human immunodeficiency virus
MesH: Medical Subject Headings
HF: Heart failure
PRISMA: Preferred Reporting Items for Systematic Reviews and Meta-analysis

## References

[1] Liang X, Shi L, Wang Y, Xiao W, Duan G, Yang H, et al. The association of hypertension with the severity and mortality of COVID-19 patients: evidence based on adjusted effect estimates. J Infect. 2020; 10.1016/j.jinf.2020.1006.1060. 10.1016/j.jinf.2020.06.060 PMC7315979 32593655

[2] Cen Y, Chen X, Shen Y, Zhang XH, Lei Y, Xu C, et al. Risk factors for disease progression in patients with mild to moderate coronavirus disease 2019-a multi-centre observational study. Clin Microbiol Infect. 2020; 10.1016/j.cmi.2020.1005.1041. 10.1016/j.cmi.2020.05.041 PMC7280135 32526275

[3] Cummings MJ, Baldwin MR, Abrams D, Jacobson SD, Meyer BJ, Balough EM (2020). Epidemiology, clinical course, and outcomes of critically ill adults with COVID-19 in New York City: a prospective cohort study. Lancet (London, England).

[4] Li X, Guan B, Su T, Liu W, Chen M, Waleed KB, Guan X, Gary T, Zhu Z (2020). Impact of cardiovascular disease and cardiac injury on in-hospital mortality in patients with COVID-19: a systematic review and meta-analysis. Heart..

[5] Borghesi A, Zigliani A, Masciullo R, Golemi S, Maculotti P, Farina D (2020). Radiographic severity index in COVID-19 pneumonia: relationship to age and sex in 783 Italian patients. Radiol Med.

[6] Jones J, Sullivan PS, Sanchez TH, Guest JL, Hall EW, Luisi N, Zlotorzynska M, Wilde G, Bradley H, Siegler AJ (2020). Similarities and Differences in COVID-19 Awareness, Concern, and Symptoms by Race and Ethnicity in the United States: Cross-Sectional Survey. J Med Int Res..

[7] Mustafa NM (2020). L AS: Characterisation of COVID-19 Pandemic in Paediatric Age Group: A Systematic Review and Meta-Analysis. J Clin Virol..

[8] Zhou F, Yu T, Du R, Fan G, Liu Y, Liu Z (2020). Clinical course and risk factors for mortality of adult inpatients with COVID-19 in Wuhan, China: a retrospective cohort study. Lancet (London, England).

[9] Robilotti EV, Babady NE, Mead PA, Rolling T, Perez-Johnston R, Bernardes M, Bogler Y, Caldararo M, Figueroa CJ, Glickman MS, Joanow A, Kaltsas A, Lee YJ, Lucca A, Mariano A, Morjaria S, Nawar T, Papanicolaou GA, Predmore J, Redelman-Sidi G, Schmidt E, Seo SK, Sepkowitz K, Shah MK, Wolchok JD, Hohl TM, Taur Y, Kamboj M (2020). Determinants of COVID-19 disease severity in patients with cancer. Nat Med..

[10] Louapre C, Collongues N, Stankoff B, Giannesini C, Papeix C, Bensa C, Deschamps R, Créange A, Wahab A, Pelletier J, Heinzlef O, Labauge P, Guilloton L, Ahle G, Goudot M, Bigaut K, Laplaud DA, Vukusic S, Lubetzki C, De Sèze J (2020). Clinical characteristics and outcomes in patients with coronavirus disease 2019 and multiple sclerosis. JAMA Neurol.

[11] Liberati A, Altman DG, Tetzlaff J, Mulrow C, Gøtzsche PC, Ioannidis JP (2009). The PRISMA statement for reporting systematic reviews and meta-analyses of studies that evaluate healthcare interventions: explanation and elaboration. BMJ.

[12] Stang A (2010). Critical evaluation of the Newcastle-Ottawa scale for the assessment of the quality of nonrandomized studies in meta-analyses. Eur J Epidemiol..

[13] Greenland S (1987). Quantitative methods in the review of epidemiologic literature. Epidemiol Rev..

[14] IntHout J, Ioannidis JP, Rovers MM, Goeman JJ (2016). Plea for routinely presenting prediction intervals in meta-analysis. BMJ Open..

[15] Borenstein M, Higgins JP, Hedges LV, Rothstein HR (2017). Basics of meta-analysis: I(2) is not an absolute measure of heterogeneity. Res Synth Methods..

[16] Begg CB, Mazumdar M (1994). Operating characteristics of a rank correlation test for publication bias. Biometrics..

[17] Egger M, Davey Smith G, Schneider M, Minder C (1997). Bias in meta-analysis detected by a simple, graphical test. BMJ.

[18] Schwarzer G, Carpenter J, Rücker G (2010). Empirical evaluation suggests Copas selection model preferable to trim-and-fill method for selection bias in meta-analysis. J Clin Epidemiol..

[19] Yu X, Sun X, Cui P, Pan H, Lin S, Han R (2020). Epidemiological and clinical characteristics of 333 confirmed cases with coronavirus disease 2019 in Shanghai, China. Transbound Emerg Dis.

[20] Zhao M, Wang M, Zhang J, Gu J, Zhang P, Xu Y (2020). Comparison of clinical characteristics and outcomes of patients with coronavirus disease 2019 at different ages. Aging..

[21] Sabri A, Davarpanah AH, Mahdavi A, Abrishami A, Khazaei M, Heydari S (2020). Novel coronavirus disease 2019: predicting prognosis with a computed tomography-based disease severity score and clinical laboratory data. Pol Arch Intern Med..

[22] Lala A, Johnson KW, Januzzi JL, Russak AJ, Paranjpe I, Richter F, Zhao S, Somani S, Van Vleck T, Vaid A, Chaudhry F, De Freitas JK, Fayad ZA, Pinney SP, Levin M, Charney A, Bagiella E, Narula J, Glicksberg BS, Nadkarni G, Mancini DM, Fuster V (2020). Prevalence and impact of myocardial injury in patients hospitalized with COVID-19 infection. JAm Coll Cardiol..

[23] Ciceri F, Castagna A, Rovere-Querini P, De Cobelli F, Ruggeri A, Galli L (2020). Early predictors of clinical outcomes of COVID-19 outbreak in Milan, Italy. Clin Immunol.

[24] Barman HA, Atici A, Sahin I, Alici G, Tekin EA, Baycan OF, et al. Prognostic significance of cardiac injury in COVID-19 patients with and without coronary artery disease. Coron Artery Dis. 2020; Publish Ahead of Print. 10.1097/MCA.0000000000000914 PMC7365584 32568741

[25] Bravi F, Flacco ME, Carradori T, Volta CA, Cosenza G, De Togni A, Martellucci CA, Parruti, Mantovani GL, Manzoli L, Shimosawa T (2020). Predictors of severe or lethal COVID-19, including angiotensin converting enzyme inhibitors and angiotensin II receptor blockers, in a sample of infected Italian citizens. PLOS ONE.

[26] Deiana G, Azara A, Dettori M, Delogu F, Vargiu G, Gessa G, Stroscio F, Tidore M, Steri G, Castiglia P (2020). Deaths in SARS-Cov-2 positive patients in Italy: the influence of underlying health conditions on lethality. Int J Environ Res Public Health..

[27] Zhang C, Qin L, Li K, Wang Q, Zhao Y, Xu B (2020). A novel scoring system for prediction of disease severity in COVID-19. Front Cell Infect Microbiol..

[28] Nie Y, Li J, Huang X, Guo W, Zhang X, Ma Y, Wang H, Qi M, Tang X, Shen X, Dai X (2020). Epidemiological and clinical characteristics of 671 COVID-19 patients in Henan Province, China. Int J Epidemiol.

[29] Hashemi N, Viveiros K, Redd WD, Zhou JC, McCarty TR, Bazarbashi AN, Hathorn KE, Wong D, Njie C, Shen L, Chan WW (2020). Impact of chronic liver disease on outcomes of hospitalized patients with COVID‐19: a multicentre United States experience. Liver Int..

[30] Lanza E, Muglia R, Bolengo I, Santonocito GO, Lisi C, Angelotti G, Morandini P, Savevski V, Politi LS, Balzarini L (2020). Quantitative chest CT analysis in COVID-19 to predict the need for oxygenation support and intubation. Euro Radiol..

[31] Zeng Z, Ma Y, Zeng H, Huang P, Liu W, Jiang M, Xiang X, Deng D, Liao X, Chen P, Chen Y (2021). Simple nomogram based on initial laboratory data for predicting the probability of ICU transfer of COVID‐19 patients: multicenter retrospective study. J Med Virol..

[32] Petrilli CM, Jones SA, Yang J, Rajagopalan H, O'Donnell LF, Chernyak Y, Tobin K, Cerfolio RJ, Francois F, Horwitz LI (2020). Factors associated with hospitalization and critical illness among 4,103 patients with COVID-19 disease in New York City. BMJ..

[33] Arshad S, Kilgore P, Chaudhry ZS, Jacobsen G, Wang DD, Huitsing K (2020). Treatment with hydroxychloroquine, azithromycin, and combination in patients hospitalized with COVID-19. IJID..

[34] San Roman JA, Uribarri A, Amat-Santos IJ, Aparisi A, Catala P, Gonzalez-Juanatey JR (2020). The presence of heart disease worsens prognosis in patients with COVID-19. Rev Esp Cardiol..

[35] Cheng B, Hu J, Zuo X, Chen J, Li X, Chen Y, Yang G, Shi X, Deng A (2020). Predictors of progression from moderate to severe coronavirus disease 2019: a retrospective cohort. Clin Microbiol Infect..

[36] Oussalah A, Gleye S, Urmes IC, Laugel E, Callet J, Barbé F, et al. Long-term ACE inhibitor/ARB use is associated with severe renal dysfunction and acute kidney injury in patients with severe COVID-19: results from a referral center cohort in the Northeast of France. Clin Infect Dis. 2020. 10.1093/cid/ciaa677 PMC7454376 32623470

[37] Kim DW, Byeon KH, Kim J, Cho KD, Lee N. The correlation of comorbidities on the mortality in patients with COVID-19: an observational study based on the Korean national health insurance big data. J Korean Med Sci. 2020;35(26). 10.3346/jkms.2020.35.e243 PMC7338208 32627443

[38] Chen J, Bai H, Liu J, Chen G, Liao Q, Yang J, Wu P, Wei J, Ma D, Chen G, Ai J, Li K (2020). Distinct clinical characteristics and risk factors for mortality in female inpatients with coronavirus disease 2019 (COVID-19): a sex-stratified, large-scale cohort study in Wuhan, China. Clin Infect Dis.

[39] Ferrante G, Fazzari F, Cozzi O, Maurina M, Bragato R, D’Orazio F, Torrisi C, Lanza E, Indolfi E, Donghi V, Mantovani R, Liccardo G, Voza A, Azzolini E, Balzarini L, Reimers B, Stefanini GG, Condorelli G, Monti L (2020). Risk factors for myocardial injury and death in patients with COVID-19: insights from a cohort study with chest computed tomography. Cardiovasc Res..

[40] Rastad H, Karim H, Ejtahed HS, Tajbakhsh R, Noorisepehr M, Babaei M (2020). Risk and predictors of in-hospital mortality from COVID-19 in patients with diabetes and cardiovascular disease. Diabetol Metab Syndr..

[41] Hwang JM, Kim JH, Park JS, Chang MC, Park D (2020). Neurological diseases as mortality predictive factors for patients with COVID-19: a retrospective cohort study. Neurol Sci..

[42] Grasselli G, Greco M, Zanella A, Albano G, Antonelli M, Bellani G, Bonanomi E, Cabrini L, Carlesso E, Castelli G, Cattaneo S, Cereda D, Colombo S, Coluccello A, Crescini G, Molinari AF, Foti G, Fumagalli R, Iotti GA, Langer T, Latronico N, Lorini FL, Mojoli F, Natalini G, Pessina CM, Ranieri VM, Rech R, Scudeller L, Rosano AR, Storti E, Thompson BT, Tirani M, Villani PG, Pesenti A, Cecconi M, Agosteo E, Albano G, Albertin A, Alborghetti A, Aldegheri G, Antonini B, Barbara E, Bardelloni G, Basilico S, Belgiorno N, Bellani G, Beretta E, Berselli A, Bianciardi L, Bonanomi E, Bonazzi S, Borelli M, Bottino N, Bronzini N, Brusatori S, Cabrini L, Capra C, Carnevale L, Castelli G, Catena E, Cattaneo S, Cecconi M, Celotti S, Cerutti S, Chiumello D, Cirri S, Citerio G, Colombo S, Coluccello A, Coppini D, Corona A, Cortellazzi P, Costantini E, Covello DR, Crescini G, De Filippi G, Poli MD, Dughi P, Fieni F, Florio G, Molinari AF, Foti G, Fumagalli R, Galletti M, Gallioli GA, Gay H, Gemma M, Gnesin P, Grasselli G, Greco S, Greco M, Grosso P, Guatteri L, Guzzon D, Iotti GA, Keim R, Langer T, Latronico N, Lombardo A, Lorini FL, Mamprin F, Marino G, Marino F, Merli G, Micucci A, Militano CR, Mojoli F, Monti G, Muttini S, Nadalin S, Natalini G, Perazzo P, Perego GP, Perotti L, Pesenti A, Pessina CM, Petrucci N, Pezzi A, Piva S, Portella G, Protti A, Racagni M, Radrizzani D, Raimondi M, Ranucci M, Rech R, Riccio M, Rosano A, Ruggeri P, Sala G, Salvi L, Sebastiano P, Severgnini P, Sigurtà D, Stocchetti N, Storti E, Subert M, Tavola M, Todaro S, Torriglia F, Tubiolo D, Valsecchi R, Villani PG, Viola U, Vitale G, Zambon M, Zanella A, Zoia E (2020). Risk factors associated with mortality among patients with COVID-19 in intensive care units in Lombardy, Italy. JAMA Int Med.

[43] Deng P, Ke Z, Ying B, Qiao B, Yuan L (2020). The diagnostic and prognostic role of myocardial injury biomarkers in hospitalized patients with COVID-19. Int J Clin Chem..

[44] Al‐Salameh A, Lanoix AP, Bennis Y, Andrejak C, Brochot E, Deschasse G, et al. Characteristics and outcomes of ‐19 in hospitalized patients with and without diabetes. Diab/Metab Res Rev. 2021;37(3). 10.1002/dmrr.3388 PMC7404605 32683744

[45] Atkins JL, Masoli JAH, Delgado J, Pilling LC, Kuo CL, Kuchel GA, Melzer DA, Newman AB (2020). Preexisting comorbidities predicting COVID-19 and mortality in the UK biobank community cohort. J Gerontol Ser A.

[46] Yao JS, Paguio JA, Dee EC, Tan HC, Moulick A, Milazzo C, Jurado J, Penna ND, Celi LA (2021). The minimal effect of zinc on the survival of hospitalized patients with COVID-19. Chest..

[47] Pinto C, Berselli A, Mangone L, Damato A, Iachetta F, Foracchia M, Zanelli F, Gervasi E, Romagnani A, Prati G, Lui S, Venturelli F, Vicentini M, Besutti G, De Palma R, Rossi PG (2020). SARSCoV-2 positive hospitalized cancer patients during the Italian Outbreak: the cohort study in Reggio Emilia. Biology..

[48] Chilimuri S, Sun H, Alemam A, Mantri N, Shehi E, Tejada J (2020). Predictors of mortality in adults admitted with COVID-19: retrospective cohort study from New York City. Western J Emerg Med..

[49] Lian J, Jin C, Hao S, Zhang X, Yang M, Jin X (2020). High neutrophil-to-lymphocyte ratio associated with progression to critical illness in older patients with COVID-19: a multicenter retrospective study. Aging..

[50] Zhao Z, Chen A, Hou W, Graham JM, Li H, Richman PS (2020). Prediction model and risk scores of ICU admission and mortality in COVID-19. PloS one..

[51] Wang L, Foer D, Bates DW, Boyce JA, Zhou L (2020). Risk factors for hospitalization, intensive care, and mortality among patients with asthma and COVID-19. J Allergy Clin Immunol..

[52] Garcia-Azorin D, Martinez-Pias E, Trigo J, Hernandez-Perez I, Valle-Penacoba G, Talavera B (2020). Neurological comorbidity is a predictor of death in Covid-19 disease: a cohort study on 576 patients. Front Neurol..

[53] Alkhatib AL, Kreniske J, Zifodya JS, Fonseca V, Tahboub M, Khatib J, Denson JL, Lasky JA, Lefante JJ, Bojanowski CM (2020). BMI is associated with coronavirus disease 2019 intensive care unit admission in African Americans. Obesity..

[54] Hernández-Galdamez DR, González-Block MÁ, Romo-Dueñas DK, Lima-Morales R, Hernández-Vicente IA, Lumbreras-Guzmán M, Méndez-Hernández P (2020). Increased risk of hospitalization and death in patients with COVID-19 and pre-existing noncommunicable diseases and modifiable risk factors in Mexico. Arch Med Res.

[55] Bellmann-Weiler R, Lanser L, Barket R, Rangger L, Schapfl A, Schaber M, Fritsche G, Wöll E, Weiss G (2020). Prevalence and predictive value of anemia and dysregulated iron homeostasis in patients with COVID-19 infection. J Clin Med.

[56] Berenguer J, Ryan P, Rodríguez-Baño J, Jarrín I, Carratalà J, Pachón J (2020). Characteristics and predictors of death among 4035 consecutively hospitalized patients with COVID-19 in Spain. Clin Microbiol Infect..

[57] Gottlieb M, Sansom S, Frankenberger C, Ward E, Hota B, Jang T (2020). Clinical course and factors associated with hospitalization and critical illness among COVID‐19 patients in Chicago, Illinois. Acad Emerg Med.

[58] Agarwal S, Schechter C, Southern W, Crandall JP, Tomer Y (2020). Preadmission diabetes-specific risk factors for mortality in hospitalized patients with diabetes and coronavirus disease 2019. Diabetes Care.

[59] Shang Y, Liu T, Wei Y, Li J, Shao L, Liu M (2020). Scoring systems for predicting mortality for severe patients with COVID-19. E Clin Med..

[60] Shi S, Qin M, Shen B, Cai Y, Liu T, Yang F, Gong W, Xu L, Liang J, Zhao Q, He H, Yang B, Huang C (2020). Association of cardiac injury with mortality in hospitalized patients with COVID-19 in Wuhan, China. JAMA Cardiol.

[61] Posso M, Comas M, Román M, Domingo L, Louro J, González C, Sala M, Anglès A, Cirera I, Cots F, Frías V-M, Gea J, Güerri-Fernández R, Masclans JR, Noguès X, Vázquez O, Villar-García J, Horcajada JP, Pascual J, Castells X (2020). Comorbidities and mortality in patients with COVID-19 aged 60 years and older in a University Hospital in Spain. Arch Bronconeumol.

[62] Shu L, Wang X, Li M, Chen X, Ji N, Shi L, Wu M, Deng K, Wei J, Wang X, Yang C, Yan J, Feng G (2021). Clinical characteristics of moderate COVID‐19 patients aggravation in Wuhan Stadium Cabin Hospital: A 571 cases of retrospective cohort study. J Med Virol.

[63] Parra-Bracamonte GM, Lopez-Villalobos N, Parra-Bracamonte FE (2020). Clinical characteristics and risk factors for mortality of patients with COVID-19 in a large data set from Mexico. Ann Epidemiol.

[64] Pablos JL, Galindo M, Carmona L, Lledó A, Retuerto M, Blanco R, Gonzalez-Gay MA, Martinez-Lopez D, Castrejón I, Alvaro-Gracia JM, Fernández DF, Mera-Varela A, Manrique-Arija S, Vázquez NM, Fernandez-Nebro A (2020). Clinical outcomes of hospitalised patients with COVID-19 and chronic inflammatory and autoimmune rheumatic diseases: a multicentric matched cohort study. Ann Rheum Dis.

[65] Zhang B, Liu S, Lu Z, Dong Y, Zhang S (2021). Previous cardiovascular surgery significantly increases the risk of developing critical illness in patients with COVID-19. J Infect.

[66] Fox T, Ruddiman K, Lo KB, Peterson E, DeJoy R, Salacup G, Pelayo J, Bhargav R, Gul F, Albano J, Azmaiparashvili Z, Anastasopoulou C, Patarroyo-Aponte G (2021). The relationship between diabetes and clinical outcomes in COVID-19: a single-center retrospective analysis. Acta Diabetol.

[67] Vena A, Giacobbe DR, Di Biagio A, Mikulska M, Taramasso L, De Maria A, Ball L, Brunetti I, Loconte M, Patroniti NA, Robba C, Delfino E, Dentone C, Magnasco L, Nicolini L, Toscanini F, Bavastro M, Cerchiaro M, Barisione E, Giacomini M, Mora S, Baldi F, Balletto E, Berruti M, Briano F, Sepulcri C, Dettori S, Labate L, Mirabella M, Portunato F, Pincino R, Russo C, Tutino S, Pelosi P, Bassetti M, Alessandrini A, Camera M, Delfino E, De Maria A, Dentone C, Di Biagio A, Dodi F, Ferrazin A, Mazzarello G, Mikulska M, Nicolini L, Toscanini F, Giacobbe DR, Vena A, Taramasso L, Balletto E, Portunato F, Schenone E, Rosseti N, Baldi F, Berruti M, Briano F, Dettori S, Labate L, Magnasco L, Mirabella M, Pincino R, Russo C, Sarteschi G, Sepulcri C, Tutino S, Pontremoli R, Beccati V, Casciaro S, Casu M, Gavaudan F, Ghinatti M, Gualco E, Leoncini G, Pitto P, Salam K, Gratarola A, Bixio M, Amelia A, Balestra A, Ballarino P, Bardi N, Boccafogli R, Caserza F, Calzolari E, Castelli M, Cenni E, Cortese P, Cuttone G, Feltrin S, Giovinazzo S, Giuntini P, Natale L, Orsi D, Pastorino M, Perazzo T, Pescetelli F, Schenone F, Serra MG, Sottano M, Tallone R, Amelotti M, Majabò MJ, Merlini M, Perazzo F, Ahamd N, Barbera P, Bovio M, Campodonico P, Collidà A, Cutuli O, Lomeo A, Fezza F, Gentilucci N, Hussein N, Malvezzi E, Massobrio L, Motta G, Pastorino L, Pollicardo N, Sartini S, Vacca P, Virga V, Porto I, Bezante G, Bona RD, La Malfa G, Valbusa A, Ad VG, Barisione E, Bellotti M, Teresita A’, Blanco A, Grosso M, Piroddi MG, Moscatelli P, Ballarino P, Caiti M, Cenni E, Giuntini P, Magnani O, Sukkar S, Cogorno L, Gradaschi R, Guiddo E, Martino E, Pisciotta L, Cavagliere B, Cristina R, Francesca F, Garibotto G, Esposito P, Bellezza C, Harusha E, Rossi F, Arboscello E, Arzani L, De Mattei L, Spadaro M, Passalacqua G, Bagnasco D, Braido F, Riccio A, Tagliabue E, Gustavino C, Ferraiolo A, Monacelli F, Mahmoud M, Tagliafico L, Napolitano A, Fiorio M, Pizzonia M, Giannotti C, Nencioni A, Giuffrida S, Rosso N, Morando A, Papalia R, Passerini D, Tiberio G, Orengo G, Battaglini A, Ruffoni S, Caglieris S (2020). Clinical characteristics, management and in-hospital mortality of patients with coronavirus disease 2019 in Genoa, Italy. Clin Microbiol Infect.

[68] Ng JH, Hirsch JS, Wanchoo R, Sachdeva M, Sakhiya V, Hong S, et al. Outcomes of patients with end-stage kidney disease hospitalized with COVID-19. Kidney Int. 2020. 10.1016/j.kint.2020.1007.1030. 10.1016/j.kint.2020.07.030 PMC7428720 32810523

[69] He F, Luo Q, Lei M, Fan L, Shao X, Huang G (2020). Risk factors for severe cases of COVID-19: a retrospective cohort study. Aging..

[70] Gupta A, Madhavan MV, Poterucha TJ, DeFilippis EM, Hennessey JA, Redfors B, et al. Association between antecedent statin use and decreased mortality in hospitalized patients with COVID-19. Res Sq [Preprint]. 2020:rs.3.rs-56210. 10.1038/s41467-021-21553-1 PMC7910606 33637713

[71] Czernichow S, Beeker N, Rives-Lange C, Guerot E, Diehl JL, Katsahian S (2020). Obesity doubles mortality in patients hospitalized for severe acute respiratory syndrome coronavirus 2 in Paris hospitals, France: a cohort study on 5,795 patients. Obesity..

[72] Sisó-Almirall A, Kostov B, Mas-Heredia M, Vilanova-Rotllan S, Sequeira-Aymar E, Sans-Corrales M (2020). Prognostic factors in Spanish COVID-19 patients: a case series from Barcelona. PloS one..

[73] Brenner H, Holleczek B, Schöttker B (2020). Vitamin D insufficiency and deficiency and mortality from respiratory diseases in a cohort of older adults: Potential for limiting the death toll during and beyond the COVID-19 pandemic?. Nutrients..

[74] De Rossi N, Scarpazza C, Filippini C, Cordioli C, Rasia S, Mancinelli CR (2020). Early use of low dose tocilizumab in patients with COVID-19: a retrospective cohort study with a complete follow-up. E Clin Med..

[75] Nimkar A, Naaraayan A, Hasan A, Pant S, Durdevic M, Suarez CN (2020). Incidence and risk factors for acute kidney injury and its effect on mortality in patients hospitalized from COVID-19. Mayo Clin Proc Innov Qual Outcomes..

[76] Klang E, Soffer S, Nadkarni G, Glicksberg B, Freeman R, Horowitz C, Reich DL, Levin MA (2020). Sex differences in age and comorbidities for COVID-19 mortality in urban New York City. SN Compr Clin Med.

[77] Emami A, Javanmardi F, Akbari A, Kojuri J, Bakhtiari H, Rezaei T (2021). Survival rate in hypertensive patients with COVID-19. Clin Exper Hypertens..

[78] Liu D, Cui P, Zeng S, Wang S, Feng X, Xu S (2020). Risk factors for developing into critical COVID-19 patients in Wuhan, China: a multicenter, retrospective, cohort study. EClinicalMedicine..

[79] Rossi PG, Marino M, Formisano D, Venturelli F, Vicentini M, Grilli R, Forloni G (2020). Characteristics and outcomes of a cohort of COVID-19 patients in the Province of Reggio Emilia, Italy. PLOS ONE.

[80] Feng X, Li P, Ma L, Liang H, Lei J, Li W (2020). Clinical characteristics and short-term outcomes of severe patients with COVID-19 in Wuhan, China. Front Med.

[81] Li G, Deng Q, Feng J, Li F, Xiong N, He Q (2020). Clinical characteristics of diabetic patients with COVID-19. J Diab Res..

[82] Seiglie J, Platt J, Cromer SJ, Bunda B, Foulkes AS, Bassett IV (2020). Diabetes as a risk factor for poor early outcomes in patients hospitalized with COVID-19. Diab Care..

[83] Tural Onur S, Altın S, Sokucu SN, Fikri B, Barça T, Bolat E (2021). Could ferritin level be an indicator of COVID-19 disease mortality?. J Med Virol..

[84] Anzola GP, Bartolaminelli C, Gregorini GA, Coazzoli C, Gatti F, Mora A (2020). Neither ACEIs nor ARBs are associated with respiratory distress or mortality in COVID-19 results of a prospective study on a hospital-based cohort. Intern Emerg Med..

[85] Ioannou GN, Locke E, Green P, Berry K, O'Hare AM, Shah JA (2020). Risk factors for hospitalization, mechanical ventilation, or death among 10 131 US veterans with SARS-CoV-2 infection. JAMA Netw Open..

[86] Bahl A, Van Baalen MN, Ortiz L, Chen NW, Todd C, Milad M (2020). Early predictors of in-hospital mortality in patients with COVID-19 in a large American cohort. Intern Emerg Med..

[87] Rafi K, Patrik Brodin N, Maron MI, Guha C, Kalnicki S, Garg MK, Racine AD (2020). Association of race and ethnicity with comorbidities and survival among patients with COVID-19 at an Urban Medical Center in New York. JAMA Network Open.

[88] Jackson BR, Gold JAW, Natarajan P, Rossow J, Fanfair RN, da Silva J, et al. Predictors at admission of mechanical ventilation and death in an observational cohort of adults hospitalized with coronavirus disease 2019. Clin Infect Dis. 2020. 10.1093/cid/ciaa1459 PMC7543323 32971532

[89] Desai A, Voza G, Paiardi S, Teofilo FI, Caltagirone G, Pons MR, Aloise M, Kogan M, Tommasini T, Savevski V, Stefanini G, Angelini C, Ciccarelli M, Badalamenti S, De Nalda AL, Aghemo A, Cecconi M, Boneschi FM, Voza A (2021). The role of anti-hypertensive treatment, comorbidities and early introduction of LMWH in the setting of COVID-19: A retrospective, observational study in Northern Italy. Int J Cardiol.

[90] Wang X, Liu Z, Li J, Zhang J, Tian S, Lu S, Qi M, Ma J, Qiu B, Weiguo Dong YX (2020). Impacts of type 2 diabetes on disease severity, therapeutic effect, and mortality of patients with COVID-19. J Clin Endocrinol Metab.

[91] Solerte SB, D'Addio F, Trevisan R, Lovati E, Rossi A, Pastore I (2020). Sitagliptin treatment at the time of hospitalization was associated with reduced mortality in patients with type 2 diabetes and COVID-19: a multicenter, case-control, retrospective, observational study. Diab Care.

[92] Hayek SS, Brenner SK, Azam TU, Shadid HR, Anderson E, Berlin H (2020). In-hospital cardiac arrest in critically ill patients with covid-19: multicenter cohort study. BMJ.

[93] Chen R, Yang J, Gao X, Ding X, Yang Y, Shen Y (2020). Influence of blood pressure control and application of renin-angiotensin-aldosterone system inhibitors on the outcomes in COVID-19 patients with hypertension. J Clin Hypertens.

[94] Lee JH, Kim YC, Cho SH, Lee J, You SC, Song YG (2020). Effect of sex hormones on coronavirus disease 2019: an analysis of 5,061 laboratory-confirmed cases in South Korea. Menopause.

[95] Nachega JB, Ishoso DK, Otokoye JO, Hermans MP, Machekano RN, Sam-Agudu NA (2020). Clinical characteristics and outcomes of patients hospitalized for COVID-19 in Africa: early insights from the Democratic Republic of the Congo. Am J Trop Med Hyg..

[96] Rozaliyani A, Savitri AI, Setianingrum F, Shelly TN, Ratnasari V, Kuswindarti R (2020). Factors associated with death in COVID-19 patients in Jakarta, Indonesia: an epidemiological study. Acta Med Indones..

[97] Wang Z, Ye D, Wang M, Zhao M, Li D, Ye J (2020). Clinical features of COVID-19 patients with different outcomes in Wuhan: a retrospective observational study. BioMed Res Int..

[98] Liu L, Wang W, Yang K, Li S, Yu X, Dong C, Zhang B (2021). Glycemic control before admission is an important determinant of prognosis in patients with coronavirus disease 2019. J Diab Investig.

[99] Al Kuwari HM, Abdul Rahim HF, Abu-Raddad LJ, Abou-Samra A-B, Al Kanaani Z, Al Khal A, Al Kuwari E, Al Marri S, Al Masalmani M, Al Romaihi HE, Al Thani MH, Coyle PV, Latif AN, Owen R, Bertollini R, Butt AA (2020). Epidemiological investigation of the first 5685 cases of SARS-CoV-2 infection in Qatar, 28 February–18 April 2020. BMJ Open.

[100] Balbi M, Caroli A, Corsi A, Milanese G, Surace A, Di Marco F, Novelli L, Silva M, Lorini FL, Duca A, Cosentini R, Sverzellati N, Bonaffini PA, Sironi S (2021). Chest X-ray for predicting mortality and the need for ventilatory support in COVID-19 patients presenting to the emergency department. Eur Radiol.

[101] Calmes D, Graff S, Maes N, Frix AN, Thys M, Bonhomme O (2021). Asthma and COPD are not risk factors for ICU stay and death in case of SARS-CoV2 infection. J Allergy Clin Immunol Pract..

[102] Talavera B, García-Azorín D, Martínez-Pías E, Trigo J, Hernández-Pérez I, Valle-Peñacoba G, Simón-Campo P, de Lera M, Chavarría-Miranda A, López-Sanz C, Gutiérrez-Sánchez M, Martínez-Velasco E, Pedraza M, Sierra Á, Gómez-Vicente B, Guerrero Á, Arenillas JF (2020). Anosmia is associated with lower in-hospital mortality in COVID-19. J Neurol Sci.

[103] Zinellu A, Arru F, De Vito A, Sassu A, Valdes G, Scano V, et al. The De Ritis ratio as prognostic biomarker of in‐hospital mortality in COVID‐19 patients. Eur J Clin Investig. 2021;51(1). 10.1111/eci.13427 PMC7646002 33043447

[104] Mallow PJ, Belk KW, Topmiller M, Hooker EA (2020). Outcomes of hospitalized COVID-19 patients by risk factors: results from a United States hospital claims database. J Health Econ Outcomes Res..

[105] Abbasi B, Akhavan R, Khameneh AG, Zandi B, Farrokh D, Rad MP, et al. Evaluation of the relationship between inpatient COVID-19 mortality and chest CT severity score. Am J Emerg Med. 2020. 10.1016/j.ajem.2020.09.056 PMC7521211 33039235

[106] Craig-Schapiro R, Salinas T, Lubetzky M, Abel BT, Sultan S, Lee JR (2021). COVID-19 outcomes in patients waitlisted for kidney transplantation and kidney transplant recipients. Am J Transplant..

[107] Ryan C, Minc A, Caceres J, Balsalobre A, Dixit A, Ng BKP, et al. Predicting severe outcomes in Covid-19 related illness using only patient demographics, comorbidities and symptoms. Am J Emerg Med. 2020. 10.1016/j.ajem.2020.09.017 PMC7480533 33046294

[108] Serin I, Sari ND, Dogu MH, Acikel SD, Babur G, Ulusoy A (2020). A new parameter in COVID-19 pandemic: initial lactate dehydrogenase (LDH)/Lymphocyte ratio for diagnosis and mortality. J Infect Public Health..

[109] Cao Y, Han X, Gu J, Li Y, Liu J, Alwalid O (2020). Prognostic value of baseline clinical and HRCT findings in 101 patients with severe COVID-19 in Wuhan, China. Sci Rep.

[110] Gupta S, Coca SG, Chan L, Melamed ML, Brenner SK, Hayek SS (2021). AKI treated with renal replacement therapy in critically ill patients with COVID-19. JASN..

[111] Raparelli V, Palmieri L, Canevelli M, Pricci F, Unim B, Lo Noce C (2020). Sex differences in clinical phenotype and transitions of care among individuals dying of COVID-19 in Italy. Biol Sex Differ..

[112] Chinnadurai R, Ogedengbe O, Agarwal P, Money-Coomes S, Abdurrahman AZ, Mohammed S (2020). Older age and frailty are the chief predictors of mortality in COVID-19 patients admitted to an acute medical unit in a secondary care setting- a cohort study. BMC Geriatr..

[113] Rajter JC, Sherman MS, Fatteh N, Vogel F, Sacks J, Rajter JJ (2021). Use of ivermectin is associated with lower mortality in hospitalized patients with coronavirus disease 2019: the ivermectin in COVID nineteen study. Chest..

[114] Naaraayan A, Nimkar A, Hasan A, Pant S, Durdevic M, Elenius H (2020). End-stage renal disease patients on chronic hemodialysis fare better with COVID-19: a retrospective cohort study from the New York Metropolitan Region. Cureus..

[115] Cherri S, Lemmers DHL, Noventa S, Abu Hilal M, Zaniboni A (2020). Outcome of oncological patients admitted with COVID-19: experience of a hospital center in northern Italy. Ther Adv Med Oncol..

[116] Rodríguez-Molinero A, Gálvez-Barrón C, Miñarro A, Macho O, López GF, Robles MT (2020). Association between COVID-19 prognosis and disease presentation, comorbidities and chronic treatment of hospitalized patients. PloS one..

[117] Clift AK, Coupland CAC, Keogh RH, Hemingway H, Hippisley-Cox J (2021). COVID-19 mortality risk in down syndrome: results from a cohort study of 8 million adults. Ann Intern Med.

[118] Clift AK, Coupland CAC, Keogh RH, Diaz-Ordaz K, Williamson E, Harrison EM, Hayward A, Hemingway H, Horby P, Mehta N (2020). Living risk prediction algorithm (QCOVID) for risk of hospital admission and mortality from coronavirus 19 in adults: national derivation and validation cohort study. BMJ..

[119] Gamberini L, Tonetti T, Spadaro S, Zani G, Mazzoli CA, Capozzi C (2020). Factors influencing liberation from mechanical ventilation in coronavirus disease 2019: multicenter observational study in fifteen Italian ICUs. J Intensive Care..

[120] Omrani AS, Almaslamani MA, Daghfal J, Alattar RA, Elgara M, Shaar SH (2020). The first consecutive 5000 patients with Coronavirus Disease 2019 from Qatar; a nation-wide cohort study. BMC Infect Dis..

[121] Yahyavi A, Hemmati N, Derakhshan P, Banivaheb B, Behnagh AK, Tofighi R, TehraniYazdi A, Kabir A (2021). Angiotensin enzyme inhibitors and angiotensin receptor blockers as protective factors in COVID-19 mortality: a retrospective cohort study. Intern Emerg Med.

[122] Guisado-Vasco P, Valderas-Ortega S, Carralón-González MM, Roda-Santacruz A, González-Cortijo L, Sotres-Fernández G, Martí-Ballesteros EM, Luque-Pinilla JM, Almagro-Casado E, La Coma-Lanuza FJ, Barrena-Puertas R, Malo-Benages EJ, Monforte-Gómez MJ, Diez-Munar R, Merino-Lanza E, Comeche-Casanova L, Remirez-de-Esparza-Otero M, Correyero-Plaza M, Recio-Rodríguez M, Rodríguez-López M, Sánchez-Manzano MD, Andreu-Vázquez C, Thuissard-Vasallo IJ, María-Tomé JME-S, Carnevali-Ruiz D (2020). Clinical characteristics and outcomes among hospitalized adults with severe COVID-19 admitted to a tertiary medical center and receiving antiviral, antimalarials, glucocorticoids, or immunomodulation with tocilizumab or cyclosporine: a retrospective observational study (COQUIMA cohort). EClinicalMedicine.

[123] Izzy S, Tahir Z, Cote DJ, Al Jarrah A, Roberts MB, Turbett S (2020). Characteristics and outcomes of latinx patients with COVID-19 in comparison with other ethnic and racial groups. Open Forum Infect Dis.

[124] Chow JH, Khanna AK, Kethireddy S, Yamane D, Levine A, Jackson AM, McCurdy MT, Tabatabai A, Kumar G, Park P, Benjenk I, Menaker J, Ahmed N, Glidewell E, Presutto E, Cain S, Haridasa N, Field W, Fowler JG, Trinh D, Johnson KN, Kaur A, Lee A, Sebastian K, Ulrich A, Peña S, Carpenter R, Sudhakar S, Uppal P, Fedeles BT, Sachs A, Dahbour L, Teeter W, Tanaka K, Galvagno SM, Herr DL, Scalea TM, Mazzeffi MA (2021). Aspirin use is associated with decreased mechanical ventilation, intensive care unit admission, and in-hospital mortality in hospitalized patients with coronavirus disease 2019. Anesth Analg.

[125] Raines AM, Tock JL, McGrew SJ, Ennis CR, Derania J, Jardak CL (2021). Correlates of death among SARS-CoV-2 positive veterans: the contribution of lifetime tobacco use. Addict Behav..

[126] Ramos-Rincon JM, Buonaiuto V, Ricci M, Martín-Carmona J, Paredes-Ruíz D, Calderón-Moreno M (2021). Clinical characteristics and risk factors for mortality in very old patients hospitalized with COVID-19 in Spain. J Gerontol Ser A Biol Sci Med Sci..

[127] Zhang L, Fan T, Yang S, Feng H, Hao B, Lu Z (2020). Comparison of clinical characteristics of COVID-19 between elderly patients and young patients: a study based on a 28-day follow-up. Aging..

[128] de Souza CD, de Arruda Magalhães AJ, Lima AJ, Nunes DN, de Fátima Machado Soares É, de Castro Silva L (2020). Clinical manifestations and factors associated with mortality from COVID-19 in older adults: retrospective population-based study with 9807 older Brazilian COVID-19 patients. Geriatr Gerontol Int..

[129] Kolhe NV, Fluck RJ, Selby NM, Taal MW, Remuzzi G (2020). Acute kidney injury associated with COVID-19: a retrospective cohort study. PLOS Med.

[130] Kim TS, Roslin M, Wang JJ, Kane J, Hirsch JS, Kim EJ (2021). BMI as a risk factor for clinical outcomes in patients hospitalized with COVID-19 in New York. Obesity..

[131] Giustino G, Croft LB, Stefanini GG, Bragato R, Silbiger JJ, Vicenzi M (2020). Characterization of myocardial injury in patients with COVID-19. J Am Coll Cardiol..

[132] An C, Lim H, Kim DW, Chang JH, Choi YJ, Kim SW (2020). Machine learning prediction for mortality of patients diagnosed with COVID-19: a nationwide Korean cohort study. Sci Rep..

[133] Piazza G, Campia U, Hurwitz S, Snyder JE, Rizzo SM, Pfeferman MB (2020). Registry of arterial and venous thromboembolic complications in patients with COVID-19. J Am Coll Cardiol..

[134] Rao X, Wu C, Wang S, Tong S, Wang G, Wu G (2020). The importance of overweight in COVID-19: A retrospective analysis in a single center of Wuhan, China. Medicine.

[135] Tehrani S, Killander A, Åstrand P, Jakobsson J, Gille-Johnson P (2021). Risk factors for death in adult COVID-19 patients: frailty predicts fatal outcome in older patients. IJID..

[136] Hyman JB, Leibner ES, Tandon P, Egorova NN, Bassily-Marcus A, Kohli-Seth R (2020). Timing of intubation and in-hospital mortality in patients with coronavirus disease 2019. Crit Care Explor..

[137] Hamilton P, Hanumapura P, Castelino L, Henney R, Parker K, Kumar M (2020). Characteristics and outcomes of hospitalised patients with acute kidney injury and COVID-19. PloS one..

[138] Liu J, Zhang S, Dong X, Li Z, Xu Q, Feng H (2020). Corticosteroid treatment in severe COVID-19 patients with acute respiratory distress syndrome. J Clin Invest..

[139] Ganatra S, Dani SS, Redd R, Rieger-Christ K, Patel R, Parikh R, et al. Outcomes of COVID-19 in patients with a history of cancer and comorbid cardiovascular disease. J Natl Compr Canc Netw. 2020:1–10. 10.6004/jnccn.2020.7658 33142266

[140] Rubio-Rivas M, Corbella X, Mora-Luján JM, Loureiro-Amigo J, López Sampalo A, Yera Bergua C, et al. Predicting clinical outcome with phenotypic clusters in COVID-19 pneumonia: an analysis of 12,066 hospitalized patients from the Spanish registry SEMI-COVID-19. J Clin Med. 2020;9(11). 10.3390/jcm9113488 PMC7693215 33137919

[141] Mendes A, Serratrice C, Herrmann FR, Genton L, Périvier S, Scheffler M, Fassier T, Huber P, Jacques MC, Prendki V (2020). Predictors of in-hospital mortality in older patients with COVID-19: the COVIDAge study. J Am Med Dir Assoc.

[142] Nemer DM, Wilner BR, Burkle A, Aguilera J, Adewumi J, Gillombardo C (2021). Clinical characteristics and outcomes of non-ICU hospitalization for COVID-19 in a nonepicenter, centrally monitored healthcare system. J Hosp Med.

[143] Guo T, Shen Q, Zhou Z, Li J, Guo W, He W (2020). Combined interventions for severe novel coronavirus disease (COVID-19): experience from 350 patients. Infect Drug Resist.

[144] Hilbrands LB, Duivenvoorden R, Vart P, Franssen CFM, Hemmelder MH, Jager KJ (2020). COVID-19-related mortality in kidney transplant and dialysis patients: results of the ERACODA collaboration. Nephrol Dial Transplant.

[145] Wang F, Cao J, Yu Y, Ding J, Eshak ES, Liu K (2021). Epidemiological characteristics of patients with severe COVID-19 infection in Wuhan, China: evidence from a retrospective observational study. Int J Epidemiol.

[146] Tang O, Bigelow BF, Sheikh F, Peters M, Zenilman JM, Bennett R, Katz MJ (2020). Outcomes of nursing home COVID-19 patients by initial symptoms and comorbidity: results of universal testing of 1970 residents. J Am Med Dir Assoc.

[147] Annweiler G, Corvaisier M, Gautier J, Dubée V, Legrand E, Sacco G, Annweiler C (2020). Vitamin D supplementation associated to better survival in hospitalized frail elderly COVID-19 patients: the GERIA-COVID quasi-experimental study. Nutrients.

[148] Huang Y, Lyu X, Li D, Wang L, Wang Y, Zou W (2020). A cohort study of 676 patients indicates D-dimer is a critical risk factor for the mortality of COVID-19. PloS one..

[149] Poterucha TJ, Elias P, Jain SS, Sayer G, Redfors B, Burkhoff D (2021). Admission cardiac diagnostic testing with electrocardiography and troponin measurement prognosticates increased 30-day mortality in COVID-19. J Am Heart Assoc.

[150] Li J, Zhang Y, Wang F, Liu B, Li H, Tang G (2020). Cardiac damage in patients with the severe type of coronavirus disease 2019 (COVID-19). BMC Cardiovasc Disord.

[151] Prado-Galbarro FJ, Sanchez-Piedra C, Gamiño-Arroyo AE, Cruz-Cruz C (2020). Determinants of survival after severe acute respiratory syndrome coronavirus 2 infection in Mexican outpatients and hospitalised patients. Public Health..

[152] Shah C, Grando DJ, Rainess RA, Ayad L, Gobran E, Benson P, Neblett MT, Nookala V (2020). Factors associated with increased mortality in hospitalized COVID-19 patients. Ann Med Surg (2012).

[153] Botta M, Tsonas AM, Pillay J, Boers LS, Algera AG, Bos LDJ (2021). Ventilation management and clinical outcomes in invasively ventilated patients with COVID-19 (PRoVENT-COVID): a national, multicentre, observational cohort study. Lancet Respir Med.

[154] Di Domenico SL, Coen D, Bergamaschi M, Albertini V, Ghezzi L, Cazzaniga MM, et al. Clinical characteristics and respiratory support of 310 COVID-19 patients, diagnosed at the emergency room: a single-center retrospective study. Intern Emerg Med. 2020:1–10. 10.1007/s11739-020-02548-0 PMC7656099 33175297

[155] Ayaz A, Arshad A, Malik H, Ali H, Hussain E, Jamil B (2020). Risk factors for intensive care unit admission and mortality in hospitalized COVID-19 patients. Acute Crit Care.

[156] Hippisley-Cox J, Young D, Coupland C, Channon KM, Tan PS, Harrison DA, Rowan K, Aveyard P, Pavord ID, Watkinson PJ: Risk of severe COVID-19 disease with ACE inhibitors and angiotensin receptor blockers: cohort study including 8.3 million people. Heart 2020, 106(19):1503-1511. 10.1136/heartjnl-2020-317393 PMC7509391 32737124

[157] Tomasoni D, Inciardi RM, Lombardi CM, Tedino C, Agostoni P, Ameri P (2020). Impact of heart failure on the clinical course and outcomes of patients hospitalized for COVID-19. Results of the Cardio-COVID-Italy multicentre study. Eur J Heart Fail.

[158] Elmunzer BJ, Wolf BJ, Scheiman JM, Tierney WM, Taylor JR (2021). Association between preadmission acid suppressive medication exposure and severity of illness in patients hospitalized with COVID-19. Gastroenterology.

[159] Polverino F, Stern DA, Ruocco G, Balestro E, Bassetti M, Candelli M, et al. Comorbidities, cardiovascular therapies, and COVID-19 mortality: a nationwide, italian observational study (ItaliCO). Front Cardiovasc Med. 2020;7. 10.3389/fcvm.2020.585866 PMC7583635 33195473

[160] Sharp AL, Huang BZ, Broder B, Smith M, Yuen G, Subject C, et al. Identifying patients with symptoms suspicious for COVID-19 at elevated risk of adverse events: The COVAS score. Am J Emerg Med. 2020. 10.1016/j.ajem.2020.10.068 PMC7642742 33189516

[161] Stebbing J, Sánchez Nievas G, Falcone M, Youhanna S, Richardson P, Ottaviani S, et al. JAK inhibition reduces SARS-CoV-2 liver infectivity and modulates inflammatory responses to reduce morbidity and mortality. Sci Adv. 2021;7(1). 10.1126/sciadv.abe4724 PMC7775747 33187978

[162] Fu L, Li XY, Fei J, Xiang Y, Xiang HX, Li MD (2020). Myocardial injury at early stage and its association with the risk of death in COVID-19 patients: a hospital-based retrospective cohort study. Front Cardiovasc Med.

[163] Sheshah E, Sabico S, Albakr RM, Sultan AA, Alghamdi KS, Al Madani K (2021). Prevalence of diabetes, management and outcomes among Covid-19 adult patients admitted in a specialized tertiary hospital in Riyadh. Saudi Arabia. Diabetes Res Clin Pract.

[164] Bowe B, Cai M, Xie Y, Gibson AK, Maddukuri G, Al-Aly Z (2020). Acute kidney injury in a national cohort of hospitalized US veterans with COVID-19. Clin J Am Soc Nephrol.

[165] Cheng X, Cai G, Wen X, Gao L, Jiang D, Sun M (2020). Clinical characteristics and fatal outcomes of hypertension in patients with severe COVID-19. Aging..

[166] Neumann-Podczaska A, Chojnicki M, Karbowski LM, Al-Saad SR, Hashmi AA, Chudek J, et al. Clinical characteristics and survival analysis in a small sample of older COVID-19 patients with defined 60-day outcome. Int J Environ Res Public Health. 2020;17(22). 10.3390/ijerph17228362 PMC7698090 33198124

[167] Ken-Dror G, Wade C, Sharma S, Law J, Russo C, Sharma A (2020). COVID-19 outcomes in UK centre within highest health and wealth band: a prospective cohort study. BMJ Open..

[168] Iannelli A, Bouam S, Schneck AS, Frey S, Zarca K, Gugenheim J, et al. The impact of previous history of bariatric surgery on outcome of COVID-19. A nationwide medico-administrative French study. Obes Surg. 2020:1–9. 10.1007/s11695-020-05120-z PMC7673863 33210274

[169] Sharifpour M, Rangaraju S, Liu M, Alabyad D, Nahab FB, Creel-Bulos CM (2020). C-Reactive protein as a prognostic indicator in hospitalized patients with COVID-19. PloS one..

[170] Martins-Filho PR, Antunes de Souza Araújo A, Pereira LX, Quintans-Júnior LJ, de Souza Barboza W, Cavalcante TF, Feitosa de Souza M, de Oliveira Góes MA, Santos VS (2021). Factors associated with mortality among hospitalized patients with COVID-19: a retrospective cohort study. Am J Trop Med Hyg..

[171] Lee SG, Park GU, Moon YR, Sung K. Clinical characteristics and risk factors for fatality and severity in patients with coronavirus disease in Korea: a nationwide population-based retrospective study using the Korean Health Insurance Review and Assessment Service (HIRA) database. Int J Environ Res Public Health. 2020;17(22). 10.3390/ijerph17228559 PMC7698934 33218161

[172] Loffi M, Piccolo R, Regazzoni V, Di Tano G, Moschini L, Robba D, et al. Coronary artery disease in patients hospitalised with Coronavirus disease; 2019. (COVID-19) infection. Open Heart. 2020;7(2). 10.1136/openhrt-2020-001428 PMC7684763 33229434

[173] Grodecki K, Lin A, Razipour A, Cadet S, McElhinney PA, Chan C (2021). Epicardial adipose tissue is associated with extent of pneumonia and adverse outcomes in patients with COVID-19. Metabolism.

[174] Khan A, Althunayyan S, Alsofayan Y, Alotaibi R, Mubarak A, Arafat M, Assiri A, Jokhdar H (2020). Risk factors associated with worse outcomes in COVID-19: a retrospective study in Saudi Arabia. East Mediterr Health J.

[175] Rutten JJS, van Loon AM, van Kooten J, van Buul LW, Joling KJ, Smalbrugge M, Hertogh C (2020). Clinical suspicion of COVID-19 in nursing home residents: symptoms and mortality risk factors. J Am Med Dir Assoc.

[176] Schuelter-Trevisol F, Raimundo LJ, Soccas HD, Antunes AF, Mohr RLD, Marcon CEM (2020). Assessment of patients with COVID-19 hospitalized in southern Santa Catarina. Rev Soc Bras Med Trop.

[177] Severity of COVID-19 and survival in patients with rheumatic and inflammatory diseases: data from the French RMD COVID-19 cohort of 694 patients. Ann Rheum Dis. 2021;80(4):527-538. 10.1136/annrheumdis-2020-218310 PMC7712850 33268442

[178] Nyabera A, Lakhdar S, Li M, Trandafirescu T, Ouedraogo TS (2020). The association between BMI and inpatient mortality outcomes in older adults with COVID-19. Cureus..

[179] Ozturk S, Turgutalp K, Arici M, Odabas AR, Altiparmak MR, Aydin Z (2021). Mortality analysis of COVID-19 infection in chronic kidney disease, haemodialysis and renal transplant patients compared with patients without kidney disease: a nationwide analysis from Turkey. Nephrol Dial Transplant.

[180] Druyan A, Lidar M, Brodavka M, Levy I, Barzilai A, Pavlotsky F (2021). The risk for severe COVID 19 in patients with autoimmune and/or inflammatory diseases: first wave lessons. Dermatol Ther.

[181] Alguwaihes AM, Al-Sofiani ME, Megdad M, Albader SS, Alsari MH, Alelayan A (2020). Diabetes and Covid-19 among hospitalized patients in Saudi Arabia: a single-centre retrospective study. Cardiovasc Diabetol.

[182] Özdemir İH, Özlek B, Özen MB, Gündüz R, Çetin N, Bilge AR (2021). Hydroxychloroquine/azithromycin treatment, QT interval and ventricular arrhythmias in hospitalised patients with COVID-19. Int J Clin Pract.

[183] Gue YX, Tennyson M, Gao J, Ren S, Kanji R, Gorog DA (2020). Development of a novel risk score to predict mortality in patients admitted to hospital with COVID-19. Sci Rep.

[184] Galiero R, Pafundi PC, Simeon V, Rinaldi L, Perrella A, Vetrano E (2020). Impact of chronic liver disease upon admission on COVID-19 in-hospital mortality: findings from COVOCA study. PloS one..

[185] Rosenthal N, Cao Z, Gundrum J, Sianis J, Safo S (2020). Risk factors associated with in-hospital mortality in a US national sample of patients with COVID-19. JAMA Netw Open..

[186] Rethemiotaki I (2020). A preliminary study of coronavirus disease 2019 in China: the impact of cardiovascular disease on death risk. Arch Med Sci Atherosclerotic Dis.

[187] Stoian AP, Pricop-Jeckstadt M, Pana A, Ileanu B-V, Schitea R, Geanta M, et al. Death by SARS-CoV 2: a Romanian COVID-19 multi-centre comorbidity study. Sci Rep. 2020;10(1). 10.1038/s41598-020-78575-w PMC7730445 33303885

[188] Zhou S, Chen C, Hu Y, Lv W, Ai T, Xia L (2020). Chest CT imaging features and severity scores as biomarkers for prognostic prediction in patients with COVID-19. Ann Transl Med..

[189] Stefan G, Mehedinti AM, Andreiana I, Zugravu AD, Cinca S, Busuioc R (2021). Clinical features and outcome of maintenance hemodialysis patients with COVID-19 from a tertiary nephrology care center in Romania. Renal Fail..

[190] Ahnach M, Zbiri S, Nejjari S, Ousti F, Elkettani C (2020). C-reactive protein as an early predictor of COVID-19 severity. J Med Biochem.

[191] Eshrati B, Baradaran HR, Erfanpoor S, Mohazzab A, Moradi Y (2020). Investigating the factors affecting the survival rate in patients with COVID-19: a retrospective cohort study. Med J Islamic Repub Iran..

[192] Özyılmaz S, Ergün Alış E, Ermiş E, Allahverdiyev S, Uçar H. Assessment of the relationship between mortality and troponin I levels in hospitalized patients with the novel coronavirus (COVID-19). Medicina. 2020;56(12). 10.3390/medicina56120693 PMC7763164 33322097

[193] Tan X, Zhang S, Xu J, Zhou M, Huang Q, Duan L (2020). Comparison of clinical characteristics among younger and elderly deceased patients with COVID-19: a retrospective study. Aging..

[194] Ling SF, Broad E, Murphy R, Pappachan JM, Pardesi-Newton S, Kong M-F, Jude EB (2020). High-dose cholecalciferol booster therapy is associated with a reduced risk of mortality in patients with COVID-19: a cross-sectional multi-centre observational study. Nutrients.

[195] Zhong Y, Zhao L, Wu G, Hu C, Wu C, Xu M (2020). Impact of renin-angiotensin system inhibitors use on mortality in severe COVID-19 patients with hypertension: a retrospective observational study. J Int Med Res..

[196] Izurieta HS, Graham DJ, Jiao Y, Hu M, Lu Y, Wu Y, Chillarige Y, Wernecke M, Menis M, Pratt D, Kelman J, Forshee R (2021). Natural history of coronavirus disease 2019: risk factors for hospitalizations and deaths among >26 million US medicare beneficiaries. J Infect Dis.

[197] Burrell AJ, Pellegrini B, Salimi F, Begum H, Broadley T, Campbell LT (2021). Outcomes for patients with COVID-19 admitted to Australian intensive care units during the first four months of the pandemic. Med J Aust..

[198] Li Y, Shang K, Bian W, He L, Fan Y, Ren T, et al. Prediction of disease progression in patients with COVID-19 by artificial intelligence assisted lesion quantification. Sci Rep. 2020;10(1). 10.1038/s41598-020-79097-1 PMC7745019 33328512

[199] Caliskan T, Saylan B (2020). Smoking and comorbidities are associated with COVID-19 severity and mortality in 565 patients treated in Turkey: a retrospective observational study. Rev Assoc Med Bras (1992).

[200] Moradi EV, Teimouri A, Rezaee R, Morovatdar N, Foroughian M, Layegh P, Kakhki BR, Koupaei SRA, Ghorani V (2021). Increased age, neutrophil-to-lymphocyte ratio (NLR) and white blood cells count are associated with higher COVID-19 mortality. Am J Emerg Med.

[201] The first wave of the COVID-19 pandemic in Spain: characterisation of cases and risk factors for severe outcomes, as at 27 April 2020. Eurosurveillance. 2020;25(50) 10.2807/1560-7917.ES.2020.25.50.2001431 PMC7812423 33334400

[202] Rashidi F, Barco S, Kamangar F, Heresi GA, Emadi A, Kaymaz C (2021). Incidence of symptomatic venous thromboembolism following hospitalization for coronavirus disease 2019: prospective results from a multi-center study. Thromb Res..

[203] Chaudhri I, Koraishy FM, Bolotova O, Yoo J, Marcos LA, Taub E (2020). Outcomes associated with the use of renin-angiotensin-aldosterone system blockade in hospitalized patients with SARS-CoV-2 infection. Kidney 360.

[204] Huh K, Ji W, Kang M, Hong J, Bae GH, Lee R (2021). Association of prescribed medications with the risk of COVID-19 infection and severity among adults in South Korea. IJID..

[205] Orioli L, Servais T, Belkhir L, Laterre PF, Thissen JP, Vandeleene B (2021). Clinical characteristics and short-term prognosis of in-patients with diabetes and COVID-19: A retrospective study from an academic center in Belgium. Diab Metab Syndr..

[206] Gude-Sampedro F, Fernández-Merino C, Ferreiro L, Lado-Baleato Ó, Espasandín-Domínguez J, Hervada X (2021). Development and validation of a prognostic model based on comorbidities to predict COVID-19 severity: a population-based study. Int J Epidemiol..

[207] Monteiro AC, Suri R, Emeruwa IO, Stretch RJ, Cortes-Lopez RY, Sherman A (2020). Obesity and smoking as risk factors for invasive mechanical ventilation in COVID-19: A retrospective, observational cohort study. PloS one..

[208] Lano G, Braconnier A, Bataille S, Cavaille G, Moussi-Frances J, Gondouin B (2020). Risk factors for severity of COVID-19 in chronic dialysis patients from a multicentre French cohort. Clin Kidney J..

[209] Lanini S, Montaldo C, Nicastri E, Vairo F, Agrati C, Petrosillo N, Scognamiglio P, Antinori A, Puro V, Di Caro A, De Carli G, Navarra A, Agresta A, Cimaglia C, Palmieri F, D’Offizi G, Marchioni L, Kobinger GP, Maeurer M, Girardi E, Capobianchi MR, Zumla A, Locatelli F, Ippolito G, Ricci S (2020). COVID-19 disease—Temporal analyses of complete blood count parameters over course of illness, and relationship to patient demographics and management outcomes in survivors and non-survivors: a longitudinal descriptive cohort study. PLOS ONE.

[210] Schwartz KL, Achonu C, Buchan SA, Brown KA, Lee B, Whelan M (2020). Epidemiology, clinical characteristics, household transmission, and lethality of severe acute respiratory syndrome coronavirus-2 infection among healthcare workers in Ontario, Canada. PloS one.

[211] Sun Y, Guan X, Jia L, Xing N, Cheng L, Liu B (2021). Independent and combined effects of hypertension and diabetes on clinical outcomes in patients with COVID-19: A retrospective cohort study of Huoshen Mountain Hospital and Guanggu Fangcang Shelter Hospital. J Clin Hypertens.

[212] McGurnaghan SJ, Weir A, Bishop J, Kennedy S, Blackbourn LAK, McAllister DA (2021). Risks of and risk factors for COVID-19 disease in people with diabetes: a cohort study of the total population of Scotland. Lancet Diab Endocrinol..

[213] Cetinkal G, Kocas BB, Ser OS, Kilci H, Yildiz SS, Ozcan SN (2020). The association between chronic use of renin-angiotensin-aldosterone system blockers and in-hospital adverse events among COVID-19 patients with hypertension. Sisli Etfal Hastanesi tip bulteni..

[214] Xu M, Yang W, Huang T, Zhou J (2020). Diabetic patients with COVID-19 need more attention and better glycemic control. World J Diab..

[215] Lv Z, Lv S (2021). Clinical characteristics and analysis of risk factors for disease progression of COVID-19: a retrospective cohort study. Int J Biol Sci..

[216] Guerra Veloz MF, Cordero Ruiz P, Ríos-Villegas MJ, Del Pino BP, Bravo-Ferrer J, Galvés Cordero R (2021). Liver manifestations in COVID-19 and the influence of pre-existing liver disease in the course of the infection. Rev Esp Enferm Dig..

[217] Chen H, Guo J, Wang C, Luo F, Yu X, Zhang W, Li J, Zhao D, Xu D, Gong Q, Liao J, Yang H, Hou W, Zhang Y (2020). Clinical characteristics and intrauterine vertical transmission potential of COVID-19 infection in nine pregnant women: a retrospective review of medical records. Lancet.

[218] Badawi A, Ryoo SG (2016). Prevalence of comorbidities in the Middle East respiratory syndrome coronavirus (MERS-CoV): a systematic review and meta-analysis. IJID..

[219] Clark A, Jit M, Warren-Gash C, Guthrie B, Wang HHX, Mercer SW, Sanderson C, McKee M, Troeger C, Ong KL, Checchi F, Perel P, Joseph S, Gibbs HP, Banerjee A, Eggo RM, Nightingale ES, O'Reilly K, Jombart T, Edmunds WJ, Rosello A, Sun FY, Atkins KE, Bosse NI, Clifford S, Russell TW, Deol AK, Liu Y, Procter SR, Leclerc QJ, Medley G, Knight G, Munday JD, Kucharski AJ, Pearson CAB, Klepac P, Prem K, Houben RMGJ, Endo A, Flasche S, Davies NG, Diamond C, van Zandvoort K, Funk S, Auzenbergs M, Rees EM, Tully DC, Emery JC, Quilty BJ, Abbott S, Villabona-Arenas CJ, Hué S, Hellewell J, Gimma A, Jarvis CI (2020). Global, regional, and national estimates of the population at increased risk of severe COVID-19 due to underlying health conditions in 2020: a modelling study. Lancet Glob Health.

[220] Guan W-j, Liang W-h, Zhao Y, Liang H-r, Chen Z-s, Li Y-m, Liu X-q, Chen R-c, Tang C-l, Wang T, Chun-quan O, Li L, Chen P-y, Sang L, Wang W, Li J-f, Li C-c, Li-min O, Cheng B, Xiong S, Ni Z-y, Jie Xiang YH, Liu L, Shan H, Lei C-l, Peng Y-x, Wei L, Liu Y, Hu Y-h, Peng P, Wang J-m, Liu J-y, Chen Z, Li G, Zheng Z-j, Qiu S-q, Luo J, Ye C-j, Zhu S-y, Cheng L-l, Ye F, Li S-y, Zheng J-p, Zhang N-f, Zhong N-s, He J-x (2020). Comorbidity and its impact on 1590 patients with COVID-19 in China: a nationwide analysis. Eur Respir J.

